# Distinct endometrial protein profiles in spontaneous and stimulated cycles in women with poor ovarian response: A prospective case-crossover clinical trial

**DOI:** 10.1371/journal.pone.0338812

**Published:** 2026-05-19

**Authors:** Dzhamilyat Abdulkhalikova, Helena Ban Frangež, Tanja Burnik Papler, Martin Štimpfel, Eda Vrtačnik Bokal, Vesna Šalamun

**Affiliations:** 1 Department of Human Reproduction, Division of Obstetrics and Gynecology, University Medical Centre Ljubljana, Ljubljana, Slovenia; 2 University of Ljubljana, Faculty of Medicine, Ljubljana, Slovenia; 3 Hospital for Women’s Diseases and Obstetrics, Postojna, Slovenia; SNUH, KOREA, REPUBLIC OF

## Abstract

The window of implantation is a critical period for embryo implantation. Ovarian stimulation can disrupt endometrial receptivity, potentially through altered gene expression and downstream protein profiles. However, the impact on the endometrial proteome remains underexplored. Identifying biomarkers of endometrial receptivity may provide an opportunity to develop targeted interventions aimed at improving implantation outcomes. This prospective, case-crossover, open-label study was conducted at the Department of Human Reproduction, Division of Obstetrics and Gynecology, University Medical Centre Ljubljana, Slovenia, from September 2023 to June 2024. The study included 15 women aged <43 years with primary infertility and poor ovarian response. Endometrial samples were collected using a pipelle biopsy during the window of implantation in spontaneous and stimulated cycles and analyzed using protein microarrays targeting 1,466 proteins. Differential protein abundance was assessed using a multi-factorial linear model, including patient-specific effects as an additional factor to account for the paired case-crossover design. Effect sizes are reported as log2-fold changes with corresponding 95% confidence intervals. Differential abundance was defined a priori as |log2FC| > 0.5 with FDR-adjusted p-value < 0.05. Comparison of endometrial samples from spontaneous and stimulated cycles revealed 114 antibodies with differential abundance. Key proteins were associated with immune response (IL-8, proteins S100-A8 and S100-A9, CAMP) and extracellular matrix remodeling (MMP-9). Exploratory KEGG pathway mapping suggested involvement of immune and inflammatory pathways, including cytokine–cytokine receptor interaction and IL-17 signaling. Cluster analysis demonstrated distinct proteomic patterns, with all stimulated-cycle samples showing alterations and a subset of stimulated-cycle samples (40%) exhibiting more pronounced changes. The findings indicate that ovarian stimulation is associated with measurable alterations in the endometrial proteomic profile during the window of implantation. These changes may be relevant to biological pathways involved in endometrial receptivity and implantation. Further studies in larger cohorts are needed to validate the identified candidate markers and determine their clinical relevance for implantation outcomes. Trial registration: ClinicalTrials.gov NCT06804174.

## Introduction

The main role of the endometrium is to provide highly specific and precisely timed support for embryo implantation. For this purpose, the endometrium undergoes dynamic, hormone-regulated changes throughout the menstrual cycle.

During most of the menstrual cycle, the endometrium remains unsuitable for embryo implantation. Dramatic physiological changes are required for the endometrium to become receptive and thus capable of accepting an embryo during a short period of the secretory phase of the menstrual cycle, referred to as the “window of implantation” (WOI) [1]. The duration of WOI is primarily determined by sex steroids, which regulate the expression of locally acting growth factors, transcription factors, cytokines, and chemokines. The transition of the endometrium to a receptive state is a highly coordinated process involving epithelial and stromal cell adhesion, trophoblast invasion, and immune modulation. Successful implantation requires accurate synchronization between the developing embryo and the receptive endometrium [2].

The field of assisted reproductive technologies has evolved considerably, with notable advancements in ovarian stimulation protocols and laboratory practices since its introduction. However, embryo implantation failure remains a major limiting factor in the success of in vitro fertilization (IVF) procedures. Although the quality of the embryo is considered to be an important determinant of successful implantation, impaired endometrial receptivity and dysregulated embryo-endometrial cross-talk are also responsible for implantation failure [3]. For this reason, numerous studies have focused on identifying markers of endometrial receptivity [4]. However, progress in developing reliable prognostic tests and treatments to enhance endometrial receptivity has been limited. One contributing factor is the lack of a clear definition of what constitutes a receptive human endometrium.

Normal endometrium function relies on specific patterns of gene expression and downstream protein abundance. »Omics« techniques, which have the ability to determine changes in different molecular compartments (e.g., genomics, proteomics, metabolomics), have enabled a better understanding of endometrial physiology as well as disease [5]. The precise molecular profiles in the human endometrium under different hormonal conditions have not been fully characterized, despite extensive research. A better understanding of these profiles holds considerable promise for uncovering markers of endometrial receptivity [5].

Ovarian stimulation with gonadotropins leads to changes in hormone levels, which are believed to affect endometrial receptivity and reduce conception rates [6, 7]. Studies have shown that the endometrium is more receptive in frozen-thawed embryo transfer cycles than in cycles with ovarian stimulation [8]. High levels of estrogen and progesterone during ovarian stimulation can affect the expression of endometrial genes and proteins that are involved in the embryo implantation process [9]. It is possible that a premature rise in progesterone during the ovarian stimulation causes a premature endometrial maturation and disrupts the WOI [10].

Comparisons of endometrial gene expression in spontaneous and stimulated cycles in the same patients have shown that gonadotropin stimulation induces the activation of genes which are not normally involved in the process of endometrial receptivity. On the other hand, expression of some genes that are known to be crucial for endometrial receptivity is reduced in stimulated cycles [11–13]. Due to numerous post-transcriptional modifications, gene expression data are not a reliable source for evaluating actual protein level dynamics. While many studies have used gene expression profiles to determine endometrial receptivity, investigations evaluating protein-level changes are limited. Therefore, this study aimed to determine differences in the endometrial protein profile during the WOI between spontaneous and stimulated cycles within the same patients with the goal of identifying clinically useful markers of endometrial receptivity that could enhance the embryo implantation process.

## Materials and methods

### Study population

This prospective case-crossover clinical trial was conducted at the Department of Human Reproduction, Division of Obstetrics and Gynecology, University Medical Centre Ljubljana, Slovenia from September 2023 to June 2024. Protein profiling of endometrial samples was performed at Sciomics GmbH, Heidelberg, Germany.

**Fig 1:**
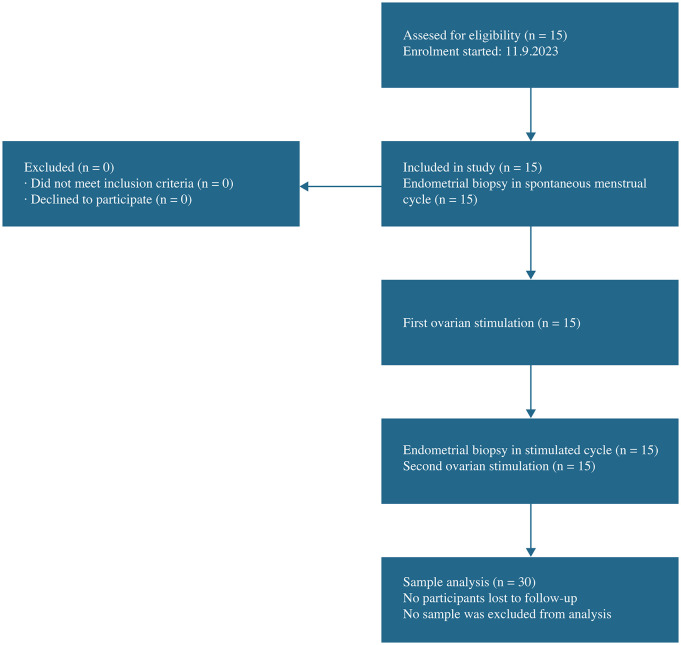
Flowchart of patient inclusion.

A total of 15 consecutive infertile patients aged <43 years, with primary infertility and poor ovarian response, were invited to participate in the study. Fig 1 shows the flowchart of patient inclusion. Patients were stratified into four groups according to the latest POSEIDON classification [14]. Representation across POSEIDON groups was limited, and subgroup sizes were small (POSEIDON 1: n = 5; POSEIDON 2: n = 3; POSEIDON 3: n = 4; POSEIDON 4: n = 3). Accordingly, the study was not powered for POSEIDON-stratified proteomic inference.

The exclusion criteria were as follows:

1. Normal or high ovarian response to gonadotropin stimulation
2. Menstrual cycle disorders
3. Severe male factor infertility
4. Preimplantation genetic testing
5. Pathological uterine findings (fibroids, endometrial polyps, adenomyosis) and tubal pathology (distal occlusion of one or both fallopian tubes with hydrosalpinx).

### Study design

To assess the protein expression profile in spontaneous menstrual cycles, the first endometrial biopsy was performed in all subjects during the WOI. Ovulation was determined using urinary LH surge test strips (visual interpretation). In all participants, the endometrial biopsy in the spontaneous cycle was performed 7 days after a clearly positive urinary LH surge test (LH + 7), thereby standardizing sampling to the mid-luteal WOI. This timing typically corresponded to cycle days 20–24, depending on individual cycle length.

The samples were collected using a plastic pipelle (Rampipella Ri.Mos.S.R.L. Mirandola, Italy), snap-frozen and stored at −80 °C until the final analysis. Following the endometrial biopsy, 4 mg of estradiol was administered to synchronize and coordinate follicular growth for a maximum of 10 days [15]. A uniform ovarian stimulation protocol was implemented in all patients during the follicular and luteal phases of the menstrual cycle. A double ovarian stimulation protocol was used, which has shown potential in patients with poor ovarian response [16–18] and requires freezing of all good-quality embryos, thereby allowing invasive endometrial biopsy during the WOI of stimulated cycles. On day 2 of the menstrual cycle, ovarian stimulation with high-dose recombinant FSH (Gonal-f 300 IU/day subcutaneously) was started according to the short antagonist stimulation protocol. On day 7 of the cycle, GnRH antagonist (Cetrorelix, Cetrotide 0.25 mg/day subcutaneously) was started. A GnRH agonist trigger (Gonapeptyl 0.1 mg subcutaneously) was administered for final oocyte maturation when at least three follicles reached 17–18 mm in diameter. Oocyte retrieval was performed 36 hours after GnRH agonist administration. No luteal phase hormonal supplementation was administered after oocyte retrieval, as all embryos were vitrified according to the freeze-all protocol required by the double stimulation approach*.* In the stimulated cycle, the endometrial biopsy was performed 7 days after the ovulation trigger, which was considered the functional equivalent of LH + 7 in the spontaneous cycle. On the same day, luteal phase ovarian stimulation was initiated using the same protocol as in the follicular phase. Fertilization, blastocyst culture, and embryo vitrification were carried out according to established laboratory methods described previously [19] and all good-quality embryos were vitrified. The warmed blastocysts were transferred in the following spontaneous or hormone-induced menstrual cycles.

The study protocol is provided as Supporting information (S2 and S3 Trial study protocols).

The laboratory outcomes related to IVF were not the focus of this manuscript and were not included in the results of this study.

### Protein microarray and statistical analysis

The primary objective of this exploratory proteomic study was to identify proteins with differential abundance in endometrial samples obtained during the WOI in spontaneous and stimulated cycles within the same patients. The estimand of interest was the within-patient log2-fold change (log2FC) in antibody signal intensity between stimulated and spontaneous cycles.

Protein profiling of 30 endometrial samples (15 paired samples) was performed using antibody-based microarrays, enabling high throughput targeting studies of a large number of proteins in different biological samples. Fifteen samples represented endometrial tissue from spontaneous menstrual cycles, while the remaining fifteen were obtained following ovarian stimulation. Proteins were extracted with scioExtract buffer (Sciomics) according to the manufacturer’s standard operating procedures, and total protein concentration was determined by bicinchoninic acid (BCA) assay. A reference sample was established by pooling equal volumes of all individual samples.

The samples were labeled at an adjusted protein concentration for 2 hours with scioDye 2 (Sciomics). The reference sample was labeled with scioDye 1 (Sciomics). After two hours, the reaction was stopped and the buffer was exchanged to phosphate-buffered saline (PBS). All labeled protein samples were stored at −20 °C until use. The samples were analyzed in a dual-color approach using a reference-based design on scioDiscover antibody microarrays (Sciomics) targeting 1,466 different proteins with 1,925 antibodies. Statistical inference was performed at the antibody (probe) level. When multiple antibodies targeted the same protein, each antibody was treated as an independent measurement, as different antibodies may recognize distinct epitopes or isoforms and yield divergent signal patterns. No probe aggregation to unique protein targets was performed. Each antibody was represented by four replicate spots on the array. The arrays were blocked with scioBlock (Sciomics) on a Hybstation 4800 (Tecan, Austria) and subsequently incubated competitively with the reference sample using a dual-color approach. Following incubation for 3 hours, the slides were thoroughly washed with 1 × PBSTT, rinsed with 0.1 × PBS and water, and subsequently dried with nitrogen [20].

Slide scanning was conducted using a Powerscanner (Tecan, Austria) with constant instrument laser power and photomultiplier tube settings. Spot segmentation and local background estimation were performed with GenePix Pro 6.0 (Molecular Devices, Union City, CA, USA). No additional background correction (e.g., normexp) was applied prior to normalization. For the scioDiscover antibody microarray platform, background levels are typically minimal due to optimized surface chemistry and blocking procedures; further background correction may increase variance when background signal is low. Acquired raw data were analyzed using the linear models for microarray data (LIMMA) package of R-Bioconductor after uploading the median signal intensities [21]. During spot segmentation, the intensity of each spot was calculated as the median of all pixel intensities within that spot. No summarization across the four replicate spots per antibody was performed prior to statistical analysis; instead, all replicate spots for each antibody and sample were retained and incorporated into the linear modeling framework. Data normalization was performed using an invariant Lowess method specifically developed for the scioDiscover antibody microarray platform [22]. This within-array normalization approach corrects for systematic intensity-dependent effects and potential dye bias inherent to dual-color microarrays. A pooled reference sample, consistently labeled with scioDye 1 across all arrays, provided a common baseline for normalization. No dye-swap experiments were performed. For differential protein abundance analysis, a multi-factorial linear model was fitted using the LIMMA package. In addition to the treatment factor (spontaneous vs. stimulated cycle), patient-specific effects were included as an additional factor in the linear model to explicitly account for the paired case-crossover design. Given the paired case-crossover design and the limited sample size, no additional covariate adjustment was applied in the proteomic models. Stable subject-level characteristics were inherently controlled by the within-subject comparison, while stimulation-related variables were considered biologically integral to the exposure of interest. Additional covariate adjustment was therefore avoided to reduce the risk of model overfitting and over-adjustment. Differential abundance between spontaneous and stimulated cycles was assessed using moderated t-statistics. All p-values were adjusted for multiple testing using the Benjamini–Hochberg false discovery rate (FDR) procedure [23]. Protein abundance differences were reported as log2FC with corresponding 95% confidence intervals derived from the paired multi-factorial linear model. Differential protein abundance was defined a priori as an absolute log2FC greater than 0.5 with a FDR-adjusted p value < 0.05. A log2FC threshold of 0.5 (corresponding to an approximately 1.4-fold change) was selected to balance statistical significance with biological relevance and to reduce the likelihood of emphasizing very small effect sizes that, although statistically significant, may not reflect meaningful biological differences in the endometrial microenvironment. Antibodies meeting both criteria were classified as differentially abundant and were presented in S5 Table. Antibodies meeting the FDR threshold but not exceeding the predefined log2FC cutoff were retained to allow transparent assessment of smaller yet statistically robust effects, and were presented separately in S6 Table. Pathway enrichment analyses and descriptive assessment of selected proteins with established relevance to endometrial receptivity were conducted as secondary exploratory analyses.

To explore whether stimulated-cycle samples exhibited distinct proteomic patterns beyond the overall stimulated versus spontaneous comparison, we performed an exploratory unsupervised hierarchical clustering analysis of antibody-level expression profiles and visualized the output as a heatmap (Fig 9). Protein signals were centered and scaled by antibody prior to clustering. Based on the hierarchical clustering dendrogram (using a two-cluster cut), samples were assigned to two clusters (Cluster 1 and Cluster 2), and cluster membership was treated as a binary exploratory grouping variable (Cluster 1 vs. Cluster 2) for subsequent post hoc comparisons with clinical and stimulation-related characteristics.

**Fig 2:**
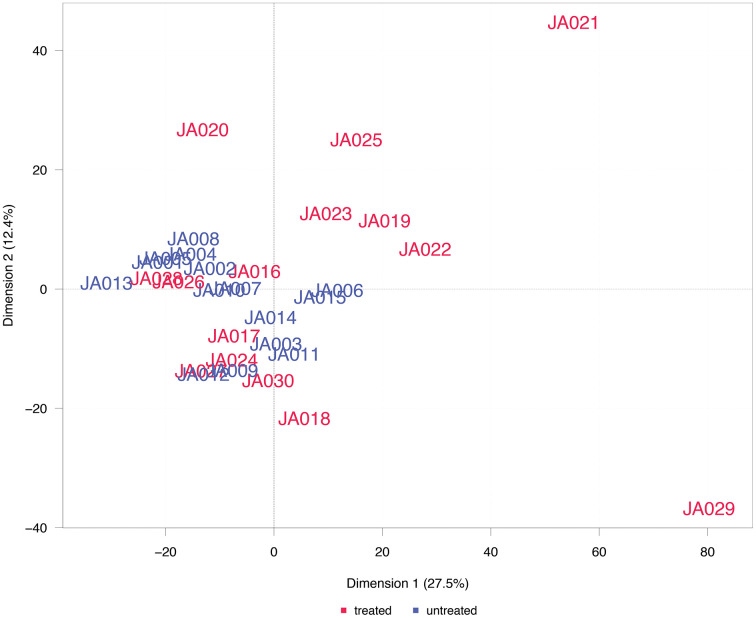
PCA of all proteins. Scatter plot displaying the first two principal components of the samples’ protein signal data using complete array data. The percentages given in the axis labels indicate the ratio of total variance explained by the respective principal component.

**Fig 3:**
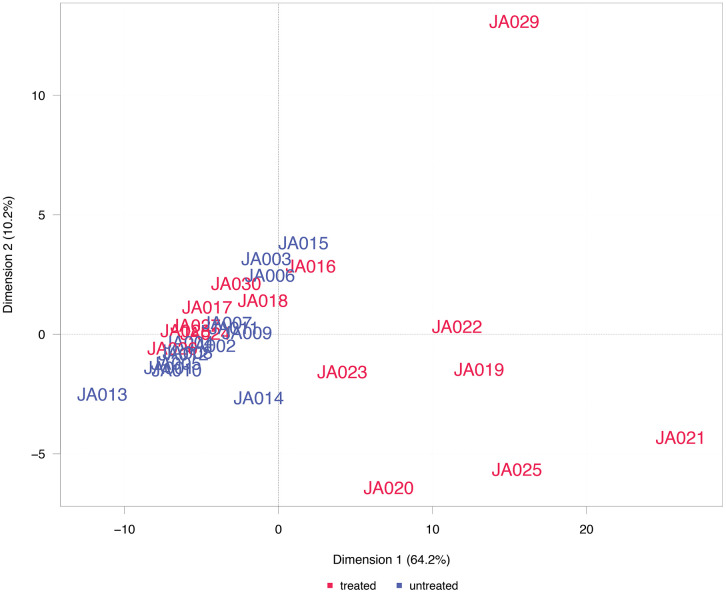
PCA of differentially abundant proteins. Scatter plot displaying the first two principal components of the samples’ protein signal data based on differentially abundant proteins. The percentages given in the axis labels indicate the ratio of total variance explained by the respective principal component.

**Fig 4:**
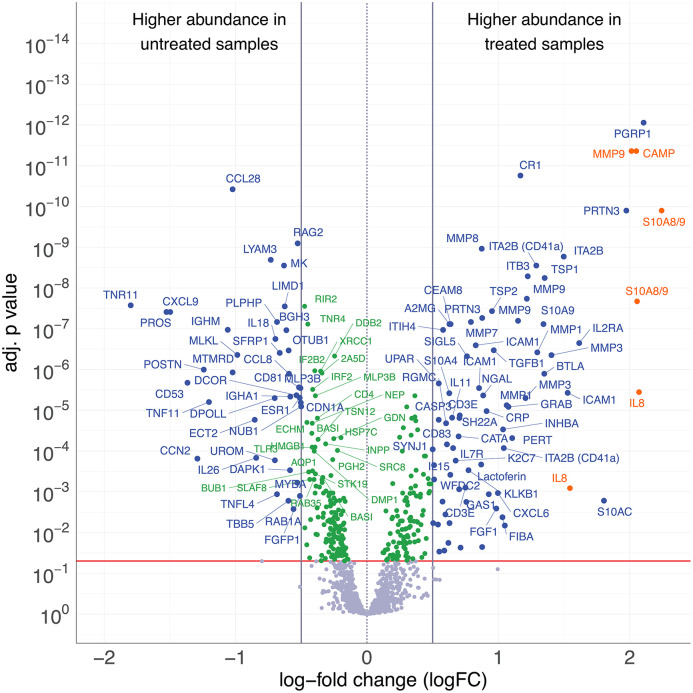
Volcano plot illustrating the differences in protein abundance between endometrial samples from spontaneous (untreated) and stimulated (treated) cycles. Protein abundance differences are expressed as log2FC, and statistical significance is shown as FDR-adjusted p-values. The horizontal red line indicates the significance threshold (FDR-adjusted p = 0.05), and the vertical lines indicate the predefined log2FC cut-offs (±0.5). Positive log2FC values indicate higher protein abundance in endometrial samples from stimulated cycles, whereas negative log2FC values indicate higher protein abundance in endometrial samples from spontaneous cycles. Proteins meeting the predefined criteria for differential abundance (|log2FC| > 0.5 and FDR-adjusted p value < 0.05) are labeled in blue. Proteins meeting the FDR-adjusted p value criterion but not exceeding the log2FC threshold are shown in green.

**Fig 5:**
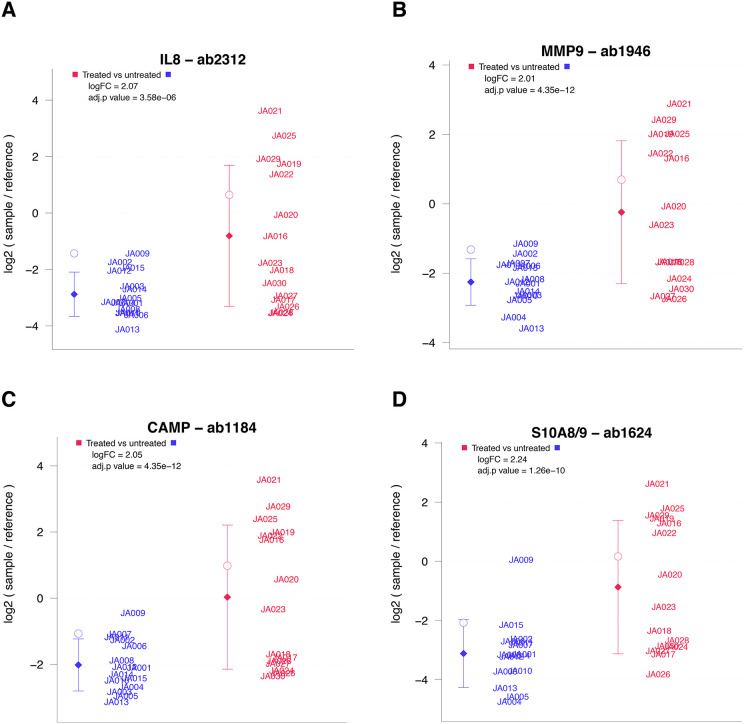
Relative levels of five proteins showing significant differential abundance in endometrial samples from spontaneous and stimulated cycles. Each sample was measured by four replicate spots per array. Diamonds indicate sample group means. Whiskers indicate one standard deviation. **A.** Interleukin-8 (IL-8); **B.** Matrix metalloproteinase-9 (MMP-9); **C.** Cathelicidin antimicrobial peptide (CAMP); **D.** Proteins S100-A8 and S100-A9.

**Fig 6:**
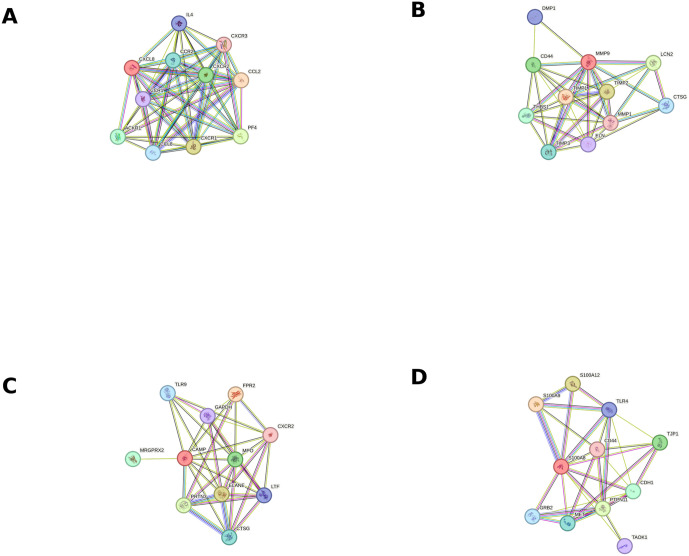
Functional associations of upregulated proteins. Colored nodes indicate direct interactions with other proteins. The threshold for establishing interaction links has been set to the highest level of confidence (0.900). The node representing the studied protein is shown in red. **A.** Interleukin-8 (IL-8); **B.** Matrix metalloproteinase-9 (MMP-9); **C.** Cathelicidin antimicrobial peptide (CAMP); **D.** Proteins S100-A8 and S100-A9.

**Fig 7:**
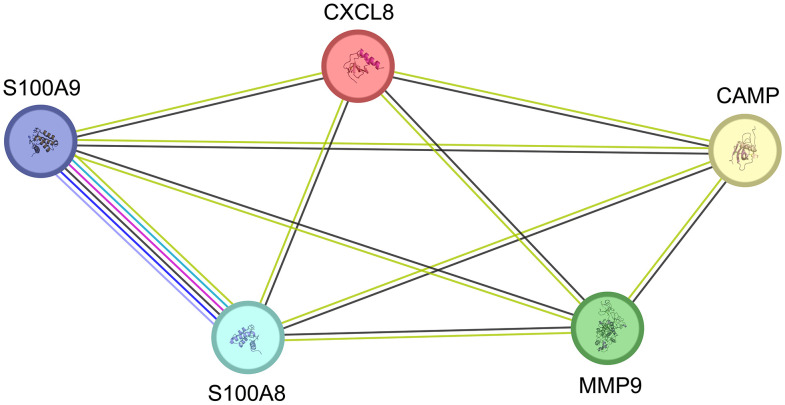
Protein-protein interactions among the proteins with the highest abundance in endometrial samples from stimulated cycles. The threshold for establishing interaction links has been set to the high level of confidence (0.700). The green line represents the simultaneous mention of related proteins in the published literature. The pink line represents the discovery of putative homologs engaging in interactions in other organisms. The black line represents the co-expression of proteins in humans or the co-expression of their putative homologs in other organisms, suggesting functional association among the proteins. Interleukin-8 (IL-8), Matrix metalloproteinase-9 (MMP-9), Cathelicidin antimicrobial peptide (CAMP), proteins S100-A8 and S100-A9.

**Fig 8:**
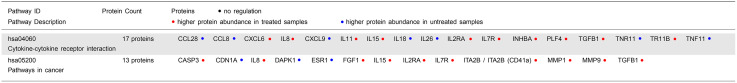
KEGG pathways with differentially abundant proteins in endometrial samples from spontaneous and stimulated cycles.

**Fig 9:**
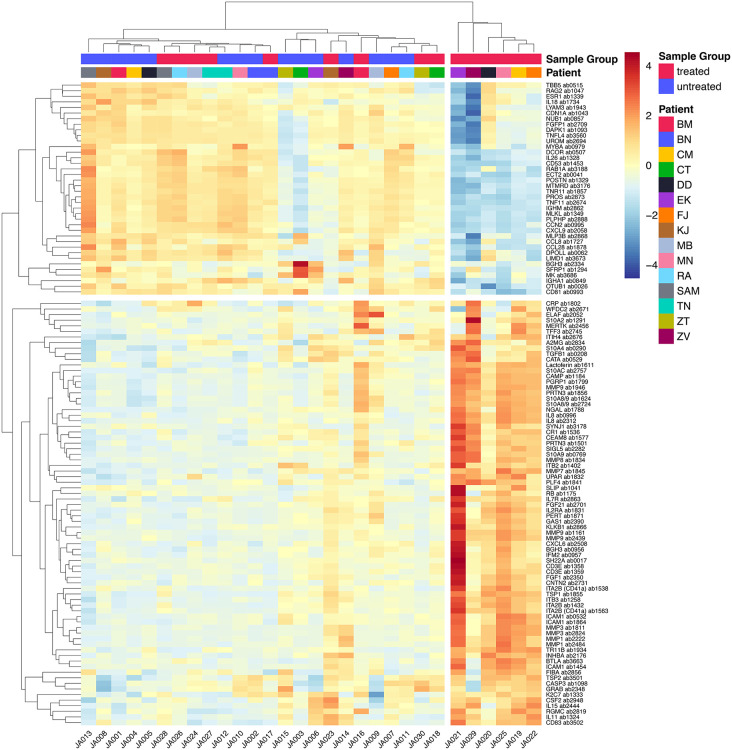
Heatmap displaying the relative protein levels of differentially abundant proteins. Values were centered and scaled by protein. Cluster 1: left cluster on the heatmap; Cluster 2: right cluster on the heatmap.

Post hoc exploratory analyses were conducted to evaluate whether cluster membership (Cluster 1 vs. Cluster 2) was associated with selected clinical and stimulation-related variables (age, BMI, stimulation duration, cumulative gonadotropin dose, smoking status, AMH, TSH, number of oocytes retrieved, number of embryos obtained, and pregnancy outcome). Continuous variables were compared using the Mann–Whitney U test and categorical variables using Fisher’s exact test. To account for multiplicity across these exploratory cluster–clinical association tests, p-values were adjusted using the Benjamini–Hochberg FDR procedure. Associations among clinical and laboratory variables were assessed using Spearman’s rank correlation and partial correlation analyses. All comparisons of clinical and laboratory characteristics were performed at the subject level, with each woman contributing a single observation. Clinical variables were not analyzed at the sample level and were therefore not duplicated across paired endometrial samples obtained from spontaneous and stimulated cycles. These analyses were considered exploratory and hypothesis-generating, given the limited sample size.

This study was reported in accordance with the CONSORT 2025 guidelines; the completed checklist is provided in the Supporting Information (S4 Checklist).

### Ethics statement

The study involving human participants was reviewed and approved by the Medical Ethics Committee of the Republic of Slovenia (0120–319/2021/3). All participants provided written informed consent to participate in this study.

The trial was registered retrospectively at ClinicalTrials.gov (NCT06804174) after the initiation of participant recruitment. This delay occurred due to a procedural oversight in recognizing registration requirements at the time the study was initiated, despite prior ethics committee approval and written informed consent for all participants. The study protocol, eligibility criteria, sampling schedule, and planned analyses were defined before data analysis, and we report all outcomes described in this manuscript transparently. Since then, we have implemented an internal pre-enrollment checklist requiring trial registration before recruitment for all prospective interventional studies, to ensure compliance with international standards in future work.

## Results

### Principal component analysis

A principal component analysis (PCA) of the protein expression data was performed on the complete array dataset (Fig 2) and on the dataset filtered for differentially abundant proteins (Fig 3). In the PCA plots, the location of the samples is defined by their first two principal components, i.e., linear combinations of protein features with the largest variance across the samples. Samples with a similar profile are located in close proximity.

### Endometrial samples from spontaneous and stimulated cycles display distinct protein profiles

A total of 114 antibodies demonstrated differential abundance between samples from spontaneous and stimulated cycles. Several proteins were significantly increased in stimulated cycles, including Interleukin-8 (IL-8; log2FC = 2.07, 95% CI 1.35–2.79; FDR-adjusted p = 3.6 × 10 ⁻ ⁶), Matrix metalloproteinase-9 (MMP-9; log2FC = 2.01, 95% CI 1.57–2.45; FDR-adjusted p = 4.4 × 10 ⁻ ¹²), Cathelicidin antimicrobial peptide (CAMP; log2FC = 2.05, 95% CI 1.60–2.50; FDR-adjusted p = 4.4 × 10 ⁻ ¹²), and proteins S100-A8 and S100-A9 (log2FC = 2.24, 95% CI 1.70–2.78; FDR-adjusted p = 1.3 × 10 ⁻ ¹⁰). The results of the statistical analysis are summarized in the volcano plot (Fig 4), while detailed information on all differentially abundant proteins, including effect sizes and confidence intervals, is provided in S5 Table. The distribution of effect sizes across all antibodies meeting the FDR criterion is provided in S6 Table, to allow comprehensive assessment of smaller but statistically reproducible changes.

### Relative protein expression levels of selected key proteins

Relative protein expression levels of the five most significantly upregulated proteins in endometrial samples from stimulated cycles compared to spontaneous cycles are presented in Fig 5.

### Functional associations of upregulated proteins suggest interactions with proteins potentially involved in endometrial receptivity

The functional associations of five upregulated proteins with significant differential abundance observed in endometrial samples from stimulated cycles were explored using the STRING v12.0 [24] and are presented in Fig 6.

### Functional associations exist among all five upregulated proteins

When investigating interactions among the most abundant proteins in endometrial samples from stimulated cycles, functional associations were found among all five proteins.

Fig 7 shows the protein-protein interactions among the proteins with the highest abundance in endometrial samples from stimulated cycles using the STRING v12 [24].

### Exploratory pathway mapping suggests involvement of immune and signaling pathways

Exploratory pathway and network analyses were performed using the STRING database to provide a descriptive overview of biological processes associated with differentially abundant antibodies. These analyses were based on curated database annotations and were not designed as independent inferential enrichment tests, nor were pathway-level multiplicity corrections applied.

KEGG pathway mapping within the STRING framework was used to contextualize the differentially abundant antibodies in relation to known biological pathways [25]. The results were presented as an overview of potentially involved biological processes, with particular emphasis on immune response and signaling pathways (Fig 8). Selected KEGG pathway maps are provided in the Supporting Information (S1 Fig).

### Exploratory hierarchical clustering suggests distinct proteomic patterns in stimulated cycles

Relative protein levels of all differentially abundant proteins are summarized in Fig 9.

Exploratory hierarchical clustering indicated differences in protein abundance patterns between endometrial samples from spontaneous and stimulated cycles. The heatmap demonstrates a clear separation of samples according to treatment exposure, indicating substantial proteomic shifts associated with ovarian stimulation.

The clustering of differentially abundant proteins identified two clusters: Cluster 1 occupies most of the left side of the heatmap and contains a mixture of endometrial samples from spontaneous and stimulated cycles, whereas Cluster 2 is located on the far right and consists exclusively of samples from stimulated cycles.

Following gonadotropin stimulation, all endometrial samples exhibited changes in protein abundance. However, in the group of samples classified as Cluster 2, stimulation induced more pronounced changes in protein abundance. To explore potential factors influencing increased protein abundance in Cluster 2, we analyzed several variables, including age, body mass index (BMI), duration of gonadotropin therapy, cumulative gonadotropin dose, pregnancy outcome, thyroid-stimulating hormone (TSH) levels, anti-Müllerian hormone (AMH) levels, smoking status, number of oocytes retrieved, and number of embryos obtained. No statistically significant associations were observed between cluster membership and the evaluated clinical variables at the nominal level. After adjustment for multiple testing using the Benjamini–Hochberg FDR procedure, no associations remained statistically significant. Spearman’s rank correlation coefficient and partial correlation analysis highlighted the strong positive association of AMH with oocyte count, as well as moderate positive associations between AMH and embryo count – findings that have been well-documented in the literature. These correlations were not specific to cluster membership.

To provide clinical context, we summarized subsequent reproductive outcomes descriptively. Pregnancies were observed only among women whose stimulated-cycle samples clustered into Cluster 2 (3/8; including one miscarriage), whereas no pregnancies occurred among women in Cluster 1 (0/7). The pregnancies observed occurred in women from different POSEIDON subgroups, suggesting that outcomes were not confined to a single POSEIDON category; however, subgroup sizes were too small for formal inference. Because clinical outcomes were not a prespecified endpoint of this exploratory study, these observations are descriptive and hypothesis-generating.

## Discussion

To our knowledge, this is the first study to report such a marked difference in protein abundance between endometrial samples from spontaneous and stimulated cycles, as well as heterogeneity among stimulated-cycle samples. This novel finding highlights the extent to which ovarian stimulation can alter the proteomic profile of the endometrium, providing new insights into the molecular changes that may influence endometrial receptivity.

We identified 114 antibodies with differential abundance between endometrial samples from spontaneous and stimulated cycles. Among these, proteins associated with immune response, inflammation, and extracellular matrix remodeling showed significant changes.

Exploratory KEGG pathway mapping provided a contextual overview of biological pathways associated with proteins showing differential abundance between endometrial samples from spontaneous and stimulated cycles, indicating a marked response to ovarian stimulation, particularly pathways related to immune response, cytokine interaction, and cancer-related signaling.

The presence of differentially abundant antibodies mapping to cytokine-related pathways is consistent with a pro-inflammatory profile. Key proteins such as IL-8, IL-18, and IL-15 implicate the recruitment of immune cells and inflammation and may reflect an association between endometrial response to stimulation and immune modulation.

Altered protein abundance observed in pathways such as “pathways in cancer” suggests that ovarian stimulation may influence cell proliferation and extracellular matrix interactions. Proteins such as MMP-9 are involved in tissue remodeling and metastasis, suggesting that ovarian stimulation may affect cellular processes that overlap with pathways also implicated in oncogenesis. In addition, we observed a well-separated cluster comprising 40% of samples from stimulated cycles (Cluster 2), with no statistically significant differences in the evaluated clinical or demographic variables between the two clusters. The separation of a subset of stimulated-cycle samples into a distinct cluster with more pronounced proteomic shifts suggests inter-individual heterogeneity in the endometrial response to ovarian stimulation. Although no statistically significant differences in measured clinical or demographic variables were detected between clusters, this should not be interpreted as evidence that no determinants exist, but rather as inconclusive given limited power and multiple-testing correction in a small cohort. Importantly, heterogeneity in receptivity-related molecular signatures is a recognized challenge, and endometrial receptivity has been proposed to represent a continuum rather than a strictly dichotomous state, which may contribute to variable molecular phenotypes within the WOI [4]. In addition, methodological guidance for endometrial ‘omics’ emphasizes that inter-individual variability, differences in hormonal and environmental exposures, and limited sample sizes can substantially influence observed molecular profiles, underscoring the need for larger, well-characterized cohorts and reproducible signatures for clinical translation [5]. Together, these considerations support interpreting Cluster 2 as a hypothesis-generating signal of variable susceptibility to stimulation-associated endometrial remodeling, warranting confirmation in adequately powered studies.

Our study supports previously established findings that ovarian stimulation significantly alters the endometrial environment during the WOI [4,9,11–13,26–28]. These changes may have implications for embryo implantation success, as endometrial receptivity is critical for embryo attachment and subsequent development.

We focused on describing selected proteins, based on their significant differential abundance as identified in the volcano plot analysis. These proteins demonstrated a strong and consistent response to ovarian stimulation. Moreover, their biological roles in critical processes such as immune modulation, inflammation, and extracellular matrix remodeling align closely with the known mechanisms influencing endometrial receptivity.

### Interleukin-8

Interleukin-8 (IL-8) is encoded by the *CXCL8* gene. It is a chemotactic factor that mediates the inflammatory response by recruiting neutrophils, basophils, and T cells to clear pathogens and protect the host from infection. It also plays an important role in neutrophil activation [29].

IL-8 is produced by a variety of cell types, including monocytes, fibroblasts, lymphocytes, epithelial cells, and endothelial cells. In the reproductive tract, it was found in the endometrium, cervix, and placenta [30]. In the human endometrium, IL-8 may modulate the recruitment of neutrophils and lymphocytes. Additionally, the amount of IL-8 mRNA in the endometrium changes throughout the menstrual cycle, suggesting that sex steroid hormones may act directly or indirectly to regulate IL-8 expression [31].

We recorded an increased IL-8 abundance in endometrial samples from stimulated cycles in our study. This finding is consistent with existing literature suggesting that ovarian stimulation may enhance the pro-inflammatory response in the endometrium [32].

IL-8 may have important implications for endometrial receptivity. Its function in the recruitment of immune cells such as neutrophils and macrophages is integral in ensuring the immune-tolerant environment required during the WOI. Edgell et al. investigated whether IL-8 levels secreted into the uterine cavity by the endometrium were altered in women with unexplained infertility compared with fertile women. The results confirmed that IL-8 was elevated in the endometrium of women with primary idiopathic infertility [32].

Fig. 6 demonstrates close functional associations between IL-8, chemokines, and their receptors. Cytokines and chemokines play a vital role during embryo implantation. They act as molecular messengers, regulating immune cells, trophoblast cells, as well as endometrial cells, facilitating the establishment of a receptive endometrium and promoting successful implantation [33]. Chemokine CCL8 has been reported to be involved in creating an appropriate environment for embryo implantation. In addition, many other chemokines have also been implicated in embryo attachment [34]. Disruption of this cytokine-chemokine network may cause implantation failure, hence establishing the great importance of these molecules as potential markers and therapeutic targets. Increased IL-8 abundance in the endometrium of stimulated cycles may indicate an altered immune response as a result of the treatment.

### Matrix metalloproteinase-9

Matrix metalloproteinase-9 (MMP-9) is encoded by the *MMP9* gene. It is primarily a cell type-specific gene, with expression especially in immune cells such as neutrophils, macrophages, and monocytes. The crucial roles of MMP-9 include the proteolytic degradation of local extracellular matrix and leukocyte migration [29]. Matrix metalloproteinases (MMPs) are a class of proteins that are thought to play a role in the breakdown of human extracellular matrix during normal physiological and pathological tissue remodeling processes such as angiogenesis, bone remodeling, wound repair, cell motility and tumor metastasis. The physiological processes of tissue remodeling during embryonic development, such as new blood vessel formation and reorganization of pre-existing vasculature, have been well characterized over the years in terms of the role played by MMPs [35]. High levels of MMP-9, expressed in response to steroids and cytokines at the maternal-fetal interface, facilitate the degradation of the endometrial extracellular matrix, loosen the intercellular junctions, and thus favor the invasion of extravillous trophoblast cells [36–38].

Some studies observed that the activity levels of MMP-2 and MMP-9 were increased in women with recurrent implantation failure compared to fertile women [39–41]. This increase may reflect an inflammatory state in the endometrium [42]. In our study we demonstrated higher MMP-9 abundance in endometrial samples from stimulated cycles.

Fig. 6 demonstrates close functional associations between MMP-9, MMP-1, and tissue inhibitors of metalloproteinases (TIMP1, TIMP2, TIMP3), which inhibit MMPs, preventing excessive extracellular matrix remodeling [43]. The balance is essential for allowing trophoblast invasion without compromising the structural integrity of the endometrial tissue. There is also a functional association between MMP-9 and CD44, which acts as a receptor for hyaluronan, collagens, and matrix metalloproteinases. The presence of this factor in endometrial cells has been confirmed by several studies [44, 45]. A recent study identified CD44 as playing a significant role in the process of embryo implantation [46]. Furthermore, reduced expression of CD44 has been shown to suppress the proliferation and decidualization of human endometrial stromal cells, which may play a pivotal role in poor endometrial receptivity in women with recurrent implantation failure [47].

### Cathelicidin antimicrobial peptide

Cathelicidin antimicrobial peptide (CAMP) is an antimicrobial peptide, encoded by the *CAMP* gene. It is a member of the cathelicidin family of peptides, which are classified as antimicrobial peptides (AMPs) and are involved in innate immunity [29]. AMPs are present in the mucosal lining of the female reproductive tract, which functions as a primary antimicrobial barrier‌‌ [48]. They are mainly secreted by epithelial cells and neutrophils in response to inflammatory or microbial stimuli. They protect the host by killing various microbes, modulating host immune properties and intervening in cellular development, tissue repair and signaling, thereby contributing to tissue homeostasis [48]. The active form of CAMP is called LL-37. Increased levels of LL-37 are present in inflamed and infected tissues. LL-37 chemoattracts T-cell leukocytes, mononuclear cells, and neutrophils [49, 50]. In addition, IL-8 stimulates neutrophils to release LL-37 which may further enhance leukocyte recruitment to the site of elevated LL-37 concentration [49]. The association between CAMP and IL-8 found in the present study is in line with previous studies suggesting that ovarian stimulation induces a pro-inflammatory state in the endometrium and may adversely impact endometrial receptivity [51].

It has been shown that LL-37 transactivates epidermal growth factor receptor (EGFR) in the airway epithelium [52]. In the endometrium, EGFR signaling is important for embryo implantation as it plays a crucial role in the maintenance of decidualization [53]. The decidua is responsible for the regulation of trophoblast invasion, modulation of the local immune response, protection from reactive oxygen species, and placental development [54]. Defective decidualization is related to adverse obstetric outcomes, such as infertility, recurrent pregnancy loss, placenta previa, preeclampsia and intrauterine growth restriction [55]. Furthermore, transactivation of EGFR by different cytokines (IL-8, TNFα, IL-1α, IL-1β, and IFNγ) seems to be important in the pathogenesis of preeclampsia [56]. In the present study, CAMP showed a higher abundance in the endometrial samples from stimulated cycles. Its association with IL-8 and EGFR via LL-37 may contribute to altered endometrial receptivity.

Fig. 6 illustrates a functional association between CAMP and the IL-8 receptor (CXCR2). The role of IL-8 in the human endometrium has already been described above. Additionally, there is a functional association between CAMP and Toll-like receptor 9 (TLR9), an important signaling molecule, implicated in local immune regulation in the human endometrium, similarly to TLR4 [57].

The proteomic differences observed between spontaneous and stimulated cycles provide important insights into the impact of ovarian stimulation on endometrial receptivity. Key findings, such as the increased abundance of immune mediators, proteolytic enzymes, and pro-inflammatory proteins, suggest a remodeling of the immune and cellular microenvironment of the endometrium, potentially limiting its receptivity. These changes, associated with ovarian stimulation, are likely driven by a complex interplay between exogenous and endogenous hormones, cytokine networks, and extracellular matrix remodeling.

### Proteins S100-A8 and S100-A9

Encoded by the *S100A8* and *S100A9* genes, these calcium- and zinc-binding proteins play a prominent role in the regulation of inflammatory processes and the immune response [29]. Calprotectin (S100A8/9) is a complex of two anionic proteins found in abundance in the cytosol of neutrophils and certain macrophages [58]. Maternal overabundance of proteins S100-A8 and S100-A9 might increase the recruitment of inflammatory leukocytes at the maternal–fetal interface potentially contributing to early pregnancy loss [59].

A functional association was observed between proteins S100-A8 and S100-A9 and Tyrosine-protein phosphatase non-receptor type 11 (*PTPN11*), a member of the protein tyrosine phosphatase family. It is a signaling molecule that regulates a variety of cellular processes including cell growth, differentiation, the mitotic cycle, and oncogenic transformation. One study demonstrated that Tyrosine-protein phosphatase non-receptor type 11 is essential for normal stromal progesterone receptor expression and epithelial–stromal interactions that occur during uterine transformation to receptivity in mice [60]. A functional association was also observed between proteins S100-A8, S100-A9, and CD44. CD44 has previously been discussed in the context of the human endometrium and embryo implantation. There is also a functional association between proteins S100-A8, S100-A9 and Toll-like receptor 4 (*TLR4*), a key activator of the innate immune response. Findings from several studies suggest that the TLR signaling molecules may contribute to local immunity in the human endometrium, suggesting their involvement in endometrial receptivity [61, 57].

### Comparison with previous transcriptomic and proteomic studies

Most previous studies examining the effects of ovarian stimulation on the endometrium have utilized transcriptomic rather than proteomic approaches. To our knowledge, only one prior study has directly examined endometrial proteomic changes following ovarian stimulation in humans. Chen et al. compared proteomic profiles of mid-secretory endometrium from patients treated with GnRH agonist or GnRH antagonist protocols versus untreated controls, identifying 362 proteins with significantly different abundance; downregulated proteins were associated with energy metabolism, and upregulated proteins were linked to cytoskeleton maintenance and complement-mediated immunity [62]. Our findings are broadly consistent with their observations of altered immune response pathways, though our case-crossover design – comparing stimulated to spontaneous cycles within the same patients – provides more direct evidence of stimulation-induced changes while controlling for inter-individual variability.

Our protein-level findings align with several gene expression changes reported in previous transcriptomic studies. Horcajadas et al. demonstrated a 2-day delay in the activation/repression of 351 genes affecting basic biological processes in the receptive endometrium following controlled ovarian stimulation [63]. Our observation of altered inflammatory and immune-related proteins is consistent with their finding that stimulation disrupts the normal temporal pattern of endometrial gene expression. Haouzi et al. reported that controlled ovarian stimulation led to disruptions in genes involved in TGF-β signaling, leukocyte transendothelial migration, and cell cycle regulation [12]. Our findings of elevated IL-8, MMP-9, and proteins S100-A8 and S100-A9 – proteins directly involved in leukocyte recruitment and migration – provide protein-level confirmation of these transcriptomic observations. Li et al. identified MMP-10 and HPSE as differentially expressed genes associated with endometrial receptivity following ovarian stimulation [64], and our observation of elevated MMP-9 protein abundance is consistent with this pattern of altered matrix metalloproteinase expression. Zhu et al. directly measured endometrial cytokine profiles and found that ovarian stimulation increased concentrations of IL-1β, IL-6, IL-7, IL-17, TNF-α, and other inflammatory mediators compared to natural cycles [65]. Our proteomic findings are consistent with this pro-inflammatory profile.

Some discrepancies exist between our findings and previous studies. Mirkin et al. reported relatively small variations in gene expression (18 genes/expressed sequence tags, fold changes between −1.55 and +3.40) and suggested these differences may not have a major functional impact on embryo implantation [66]. In contrast, we identified 114 antibodies with differential abundance, with some antibodies showing substantial fold changes (e.g., IL-8 log2FC = 2.07, representing an approximately 4.2-fold increase). These differences may be explained by several factors. First, gene expression does not always correlate with protein abundance due to post-transcriptional modifications, protein stability, and translational regulation; as Taher et al. demonstrated in a mouse model, the proteome rather than the transcriptome predicted the effects of oocyte superovulation on embryonic phenotypes, highlighting that proteomic analysis may capture functionally relevant changes not apparent at the transcriptional level [67]. Second, our study focused specifically on women with poor ovarian response, whereas most previous studies examined normal responders; women with poor ovarian response may exhibit different endometrial responses to stimulation. Third, we employed a double stimulation protocol with a GnRH agonist trigger and no luteal support, which differs from conventional protocols; as discussed below, the absence of luteal support likely contributed to the observed proteomic changes and may have accentuated inflammatory responses. Fourth, while we standardized sampling to LH + 7/GnRH agonist+7, subtle differences in the actual WOI timing between studies could contribute to variability in findings.

### Biological significance: Adaptive versus maladaptive roles

The biological significance of the elevated inflammatory proteins and matrix metalloproteinases observed in our study requires careful interpretation. While associations between elevated IL-8, MMP-9, and proteins S100-A8 and S100-A9 with implantation failure have been reported, these proteins also play essential physiological roles in normal implantation and trophoblast invasion.

IL-8 stimulates trophoblast cell migration and invasion by increasing levels of MMP-2 and MMP-9 and integrins α5 and β1 [68]. Decidual stromal cells secrete IL-8 to facilitate trophoblast invasion via the NOD1/JNK pathway [69], and uterine natural killer cells promote extravillous trophoblast invasion partially through IL-8, an effect mediated by increased MMP-2 secretion [70]. Furthermore, IL-8 in embryo culture medium has been associated with higher pregnancy rates, implantation rates, and live birth rates, suggesting it may be an independent predictor of embryo developmental potential [71]. IL-1β stimulates migration and survival of first-trimester villous cytotrophoblast cells through endometrial epithelial cell-derived IL-8 [72]. Thus, IL-8 appears to be part of a tightly regulated system where both its presence and its precise concentration are critical. The markedly elevated IL-8 we observed in stimulated cycles may represent a dysregulated inflammatory response rather than the controlled, physiological IL-8 expression required for successful implantation.

Similarly, MMP-9 has well-established physiological roles in successful implantation. Invading cytotrophoblasts increase the production and activation of MMP-9, which contributes to the invasiveness of cytotrophoblasts in vitro [73]. The simultaneous increase in the production of TIMP-3 provides a mechanism for restricting MMP-mediated invasion, and this balance between MMPs and TIMPs is critical for regulated trophoblast invasion [73]. The decidual secretome contains both pro-invasive factors (including IL-8, MMP-9) and anti-invasive factors that are antagonists to matrix metalloproteinases and/or activators of TIMPs, and this balance allows timely and regulated invasion [74]. Excessive MMP-9 activity, without corresponding increases in TIMPs, may disrupt this delicate balance and lead to either inadequate or excessive invasion.

Proteins S100-A8 and S100-A9 illustrate the complexity of inflammatory mediators in reproduction. Protein S100-A8 has distinct roles in early embryo development and in the maternal deciduum, where it is associated with vasculature and uterine natural killer cells involved in vascular remodeling [75]. However, maternal overabundance of proteins S100-A8 and S100-A9 has been associated with increased recruitment of inflammatory leukocytes at the maternal-fetal interface, resulting in early pregnancy loss [59]. This dual nature suggests that proteins S100-A8 and S100-A9, like other inflammatory mediators, may be beneficial at physiological levels but detrimental when excessively elevated.

The decidua must balance the production of pro-invasive and anti-invasive molecules to allow timely and regulated invasion [74]. Decidual secretions are enriched in immune mediators, including cytokines and chemokines that promote trophoblast invasion, alongside counter-regulatory factors that restrain invasion, indicating a balanced and tightly controlled invasion program [74]. Disruption of this balance – whether through excessive pro-invasive signaling or inadequate anti-invasive restraint – may compromise implantation success. The markedly elevated levels of inflammatory proteins we observed in stimulated cycles – particularly in the context of GnRH agonist trigger without luteal support – may represent a shift from physiological to pathological inflammation. Whether the observed proteomic changes represent maladaptive responses or exaggerated but potentially adaptive responses to altered hormonal conditions cannot be definitively determined from our study and requires further investigation with adequate luteal support and prospective correlation with implantation outcomes.

An important consideration when interpreting our findings is the absence of luteal-phase hormonal support after GnRH agonist triggering in the stimulated cycles. Although a GnRH agonist trigger induces a gonadotropin surge, in the absence of adequate supplementation it is followed by premature luteolysis and a markedly insufficient luteal phase, with reduced luteal steroid exposure (progesterone and estradiol) and shortened luteal function compared with hCG triggering [76]. In nonsupplemented cycles, progesterone levels begin to decline early after oocyte retrieval, and overall luteal progesterone and estradiol profiles remain significantly lower, consistent with profound luteal-phase insufficiency [76]. This altered hormonal milieu has measurable consequences for endometrial molecular programming. Bermejo et al. demonstrated that GnRH agonist trigger followed by “standard” luteal support limited to estradiol and progesterone (i.e., without LH/hCG activity) was associated with the largest divergence in endometrial gene expression relative to hCG trigger, with 56 differentially expressed genes; in contrast, when LH/hCG activity was added, the endometrial transcriptomic pattern more closely resembled that observed after hCG trigger, with only 30–36 differentially expressed genes [77]. Similarly, Humaidan et al. reported that endometrial gene expression in the early luteal phase differs substantially according to the mode of triggering and the presence or absence of luteal support, including genes implicated in receptivity and early implantation [78]. Collectively, these data indicate that luteal-phase endocrine conditions directly modulate endometrial gene and protein expression patterns. Accordingly, the proteomic changes observed in our study – particularly increased abundance of pro-inflammatory proteins (IL-8, proteins S100-A8 and S100-A9, CAMP) and matrix metalloproteinases (MMP-9) – may reflect not only the effects of supraphysiological ovarian stimulation, but also the downstream molecular consequences of luteal insufficiency. In line with this interpretation, suboptimal progesterone concentrations have been associated with aberrant endometrial gene expression and potentially impaired implantation [79]. Moreover, ovulation induction and inadequate luteal support have been linked to disrupted endometrial maturation, including glandulo-stromal dyssynchrony, reduced αvβ3-integrin expression, and altered pinopode morphology, all of which are consistent with impaired receptivity [80]. Finally, comparative proteomic analyses suggest that exposure to GnRH analogs may influence endometrial energy metabolism and upregulate complement-related immune pathways, broadly consistent with our findings of altered immune-related proteins [62].

This study has several important strengths. It extends beyond transcriptomic analyses by evaluating protein-level changes, which are more directly related to biological function. The prospective case-crossover design enabled direct within-patient comparison of spontaneous and stimulated cycles, thereby minimizing inter-individual variability. Differential protein abundance was assessed using a multi-factorial linear model incorporating patient-specific effects as a factor, with false discovery rate correction and reporting of effect sizes with 95% confidence intervals. The antibody-based microarray platform enabled broad proteomic profiling, and predefined analytical thresholds were applied transparently.

However, several limitations should be acknowledged. The sample size was limited, which restricted statistical power and generalizability. Statistical inference was performed at the antibody level rather than at the level of aggregated protein targets, which may complicate biological interpretation when multiple antibodies target the same protein. In addition, protein abundance was measured using an antibody-based microarray platform, which is inherently semi-quantitative and may be influenced by antibody specificity and cross-reactivity; accordingly, the highlighted targets should be regarded as candidate markers emerging from an exploratory screen rather than independently validated biomarkers. Luteal-phase hormonal markers, such as serum progesterone at the time of biopsy, were not measured, and residual variability in endometrial timing cannot be excluded. Pathway and network analyses were descriptive and hypothesis-generating, and no functional validation of the identified proteins was performed.

A major limitation is the absence of luteal-phase support after the GnRH agonist trigger in stimulated cycles. Consequently, the observed proteomic differences likely reflect the combined effects of ovarian stimulation and luteal insufficiency and may not be directly generalizable to fresh embryo transfer cycles with adequate luteal support. This hormonal context may have accentuated immune activation and extracellular matrix remodeling, thereby shaping the observed endometrial proteomic profile.

Although an exploratory descriptive comparison suggested that pregnancies occurred only among women whose stimulated-cycle samples clustered in Cluster 2, this finding must be interpreted with caution. Reproductive outcomes were achieved in subsequent transfer cycles, whereas proteomic profiling was performed in the stimulated cycle without luteal support. Therefore, the present study cannot determine whether the stimulation-associated proteomic changes observed during the WOI are detrimental, beneficial, or neutral with respect to implantation success, nor whether the apparent association between cluster membership and later reproductive outcomes reflects a causal relationship. These observations should therefore be considered hypothesis-generating only.

Finally, although estradiol used for follicular synchronization has a short half-life, residual effects on the subsequent stimulated cycle cannot be completely excluded. Future studies should incorporate standardized luteal endocrine measurements at biopsy, compare different luteal support regimens after GnRH agonist trigger, including protocols with LH/hCG activity, and relate endometrial proteomic signatures prospectively to implantation, clinical pregnancy, and live birth outcomes.

## Conclusion

Ovarian stimulation protocols involving high doses of exogenous gonadotropins have the potential to influence the endometrial environment. Such alterations may have significant implications for implantation success, as endometrial receptivity is crucial for embryo attachment and subsequent development. The present study demonstrates distinct alterations in the endometrium-specific proteome following ovarian stimulation. The findings indicate that altered protein abundance, particularly of proteins associated with the immune response and extracellular matrix remodeling, may reflect biological processes relevant to implantation biology. This study may serve as a foundation for investigating key biomarkers that could play an important role in regulating endometrial receptivity in both spontaneous and stimulated cycles.

## Supporting information

S1 Fig Selected KEGG pathways associated with proteins with differential abundance in endometrial samples from spontaneous and stimulated cycles. (TIF)

S2 Protocol Trial study protocol (English version). (DOCX)

S3 Protocol Trial study protocol (Slovenian version). (DOCX)

S4 Checklist CONSORT 2025 checklist. (DOCX)

S5 Table Proteins‌‌ with differential abundance in endometrial samples obtained during spontaneous and stimulated cycles, identified using a paired multi-factorial linear model including 95% confidence intervals. (XLSX)

S6 Table All protein differences. (XLSX)
