# Supplementary material for: Distinct endometrial protein profiles in spontaneous and stimulated cycles in women with poor ovarian response: A prospective case-crossover clinical trial
Source: PLoS One. 2026 May 19;21(5):e0338812. doi: 10.1371/journal.pone.0338812 (PMC13186353; doi:10.1371/journal.pone.0338812)
Supplement: S2 Protocol — (DOCX) [file pone.0338812.s002.docx]

APPLICATION FOR AN OPINION ON THE ETHICAL ACCEPTABILITY OF A RESEARCH PROPOSAL
 **Research title:**

"Continuous Ovarian Stimulation with Gonadotropins in Women with Poor Ovarian Response During In Vitro Fertilization Procedures"

**Research type:** prospective cross-over open-label study

**Principal investigator:** Prof. Dr. Eda Bokal Vrtačnik, MD, Senior Sdvisor (researcher code at ARRS 12177), Specialist in Gynecology and Obstetrics, Head of the Department of Human Reproduction, Division of Obstetrics and Gynaecology, University Medical Centre Ljubljana, Ljubljana, Slovenia

**Physician responsible for the safety of research subjects:** Prof. Dr. Eda Bokal Vrtačnik, MD, Senior Consultant, Specialist in Gynecology and Obstetrics

**Estimated duration of the research:** 3 years

**Research participants:**

- Prof. Dr. Borut Peterlin, Dr. med., Councilor (researcher code at ARRS 10458), Head of the Clinical Institute of Genomic Medicine
- Assoc. Prof. Dr. Nina Jančar, MD (researcher code at ARRS 25612), Specialist in Gynecology and Obstetrics, Department of Human Reproduction, Division of Obstetrics and Gynaecology, University Medical Centre Ljubljana, Ljubljana
- Dr. Martin Štimpfel, univ. dipl. biotech. (researcher code at ARRS 33917), Head of the Laboratory for Assisted Reproductive Technology, Department of Human Reproduction, Division of Obstetrics and Gynaecology, University Medical Centre Ljubljana, Ljubljana
- Dzhamilyat Abdulkhalikova, MD, Specialist in Gynecology and Obstetrics, Department of Human Reproduction, Division of Obstetrics and Gynaecology, University Medical Centre Ljubljana, Ljubljana

**Institutions:**

The research is planned at the Department of Human Reproduction, Division of Obstetrics and Gynaecology, University Medical Centre Ljubljana, where clinical examination, informed consent of the subjects, collection of uterine lavage and endometrial biopsy, infertility treatment and analysis of the obtained results will take place. The in vitro fertilization procedure and further cultivation of embryos, biopsy of the trophoblast fragment, vitrification of embryos and collection of culture medium for non-invasive examination will take place in the Laboratory for Assisted Reproductive Technology, Department of Human Reproduction, Division of Obstetrics and Gynaecology, University Medical Centre Ljubljana.

- A non-invasive method for determining the chromosomal status of an embryo after DNA isolation from the culture medium will be carried out at the Clinical Institute of Genomic Medicine, UKC Ljubljana
- Protein profiling of uterine lavages and endometrial biopsies will be performed at the Sciomics GmbH laboratory in Heidelberg (Germany).

Full title of the research: **"Continuous ovarian stimulation with gonadotropins in women with reduced ovarian response in in vitro fertilization procedures."**

1. Scientific starting points and presentation of the problem

Despite scientific and technological advances in the treatment of infertility by in vitro fertilization (IVF), we still encounter the problem of poor ovarian response (POR). These patients do not represent a homogeneous group and their prognosis depends largely on the age of the patient and the number of oocytes retrieved.(1) Data from the literature indicate that reduced response occurs in 9-24% of infertile women.(2) The prevalence increases particularly with the postponement of pregnancy into the late thirties or even forties. In approximately half of these patients, the cause is unknown.(3) While the reduction in the number of antral follicles due to aging is a well-known and explained phenomenon (3), much less is known about the genetic and chromosomal etiology and about metabolic, enzymatic, iatrogenic, toxic, autoimmune or inflammatory causes.(3)

There are many definitions for women with Ovarian Cysts. Many ovarian stimulation protocols are used to increase the live birth rate.(4)

Recently, the POSEIDON classification (Patient-Oriented Strategies Encompassing Individualized Oocyte Number) has been most frequently used to accurately define women with POI. (5) In fact, the number of retrieved oocytes and the age of the woman have the greatest influence on the success of conception.(6,7) Therefore, predicting ovarian response through individual stimulation planning is very important, especially in patients with POR. The number of antral follicles and serum AMH concentration are the most successful predictors of ovarian reserve and, consequently, the response to gonadotropin stimulation.(8)

There is currently insufficient evidence to support the superiority of any of the various known induction protocols in women with POR. It is known that the live birth rate is lower in these women compared to infertile women with normal or increased response.(9)

The realization that the recruitment of a cohort of follicles does not occur only in the follicular phase, but 2 to 3 times in one menstrual cycle, i.e. also in the luteal phase (10), leads to the idea that with continuous stimulation, a larger number of oocytes and embryos could be obtained in one menstrual cycle. The embryos would then be frozen and transferred in subsequent cycles, as we know that the pregnancy rate with fresh or thawed embryos is comparable. The first experience with double stimulation was described by Kuang et al., who reported the same number and developmental potential of oocytes obtained in the follicular and luteal phases.(11) During the first wave of the COVID-19 epidemic, we also did this at the Department of Human Reproduction, Division of Obstetrics and Gynaecology, University Medical Centre Ljubljana in older women and obtained even slightly more cells in the luteal phase compared to the follicular phase.

Given that successful fertilization requires the synchronous interaction of the receptive endometrium and high-quality euploid embryos, current research is increasingly focused on analyzing the protein profile in the endometrium with the aim of identifying and determining the optimal implantation window.

It is hypothesized that exposure to ovarian stimulation with gonadotropins and the resulting altered hormone levels impair endometrial receptivity, which reduces pregnancy rates, particularly with poorer quality embryos.(12,13) There is increasing evidence that the endometrium in thawed embryo transfer cycles is more receptive compared to the endometrium after ovarian stimulation.(14) Hormonal regulation plays an important role in endometrial receptivity, and high levels of estrogen and progesterone may affect the expression of genes and proteins involved in the process of embryo implantation .(15) Asynchrony between the embryo and the non-receptive endometrium may be a consequence of ovarian stimulation, which causes a premature increase in progesterone and consequently premature maturation of the endometrium, disrupting the implantation window period.(16)

The Act on Infertility Treatment and Assisted Reproductive Technology, in Article 31, stipulates that genetic testing of an early embryo may only be performed in cases of risk of a serious hereditary disease or if this is necessary for the success of the biomedically assisted fertilization procedure. In Slovenia, these procedures have been performed since 2004, and only in cases of risk of transmitting a serious hereditary disease to offspring. We do not perform such tests with the aim of improving the success of IVF procedures, as it is not yet completely clear on a global scale in which cases this would make sense. For the time being, it seems that they are particularly suitable for older women, as this can reduce the incidence of miscarriage and shorten the time to conception.(17) Preimplantation genetic testing (PGT) procedures, which are performed with the aim of preventing the birth of a child with a serious hereditary disease, can be further and more precisely divided into PGT-M (monogenic defects), in which embryos are tested to prevent the transmission of monogenic diseases, and PGT-SR (structural rearrangements), in which embryos are examined to prevent the transmission of structural chromosomal abnormalities. The method of examination, which attempts to improve the success of IVF procedures, is called PGT-A (aneuploidy); in this method, embryos are examined for the number of chromosomes or the presence of aneuploidy.

For genetic examination of the embryo, it is necessary to biopsy it. The biopsy can be performed at the stage of the low-cell embryo on the third day of development or at the blastocyst stage on the 5/6th day of development. The embryo on the third day of development normally has 6-8 cells, which represents a limitation in the amount of DNA. When biopsying at this stage of development, 1 or a maximum of 2 cells (blastomeres) are removed from the embryo. Research shows that removing 1-2 cells can hypothetically negatively affect the further development and implantation potential of the embryo. Another disadvantage of this approach is that it is not possible to reliably determine mitotic errors that lead to mosaicism. The advantage of the procedure is that it allows the separation of meiotic errors or mutations from both parents.

An approach that provides more reliable results is an embryo biopsy at the blastocyst stage. At that time, the embryo usually has around 100 cells, and the part of the embryo from which the fetus develops (embryoblast) and the part of the embryo from which the placenta develops (trophoblast) can already be distinguished. The biopsy is performed by cutting off a small part of the trophoblast (around 5-10 cells) with the help of a laser , and the embryo is then frozen in liquid nitrogen for the duration of the genetic test . Another advantage is that only embryos with the potential for implantation are biopsied. The disadvantage of this method is that mosaicism may occur in a certain proportion, which is less problematic than in the case of biopsy of low-cell embryos. Although research results show that PGT procedures do not have a negative impact on the health and development of children, due to the relatively invasive intervention in the embryo, concerns constantly arise that such procedures could have a negative impact on the health of children. (18)

, less invasive and non-invasive procedures for obtaining genetic material from the embryo are constantly being sought , as they could avoid all concerns about direct intervention in it. A less invasive procedure than a blastocyst biopsy is a blastocoel biopsy, which involves aspirating fluid from the blastocoel, which also contains the embryo's DNA, and the blastocyst collapses during this procedure. This procedure is sometimes even used in clinical practice before embryo vitrification, as it is said to improve the survival of the blastocyst, although it does involve some intervention in the embryo. A completely non-invasive procedure is genetic analysis of the culture medium in which the embryos are grown. This is possible because embryos secrete parts of nuclear and mitochondrial DNA during development, which can be detected with modern molecular methods and then determined the genetic status of the embryo in terms of the correct number of chromosomes. An abnormal number of chromosomes, even if the embryo successfully implants , usually leads to miscarriage, which exposes the patient to unnecessary stress and also prolongs the time until a possible new pregnancy. All of the above is very unfavorable for patients in the late reproductive period or with impending premature ovarian failure.

According to data from the our clinic (19) and the results of a recently published Dutch study (20), POR does not pose a major problem in younger patients, as the live birth rate after IVF procedures is almost the same as in well-responding patients. However, the treatment of older patients with POR poses a major challenge.

2. Review and analysis of previous research and relevant literature

The most successful “treatment” for patients with POR after unsuccessful IVF procedures is the use of donated eggs. The live birth rate for patients with POR ranges from 1 to 10% per cycle, and the live birth rate with donated eggs is between 50 and 70%.(3) The decision to use donated eggs is often difficult for couples to accept, especially when pregnancy with their own eggs can still be expected, although with a very low probability. Therefore, patients are also prepared to repeat the procedures several times. Because of the above, a great challenge for reproductive gynecologists is to find the best or most effective protocol for ovarian stimulation in IVF procedures for patients with POR. Numerous studies conducted so far have not led to uniform recommendations and decisions.(3)

A comparison of ovarian stimulation with gonadotropins in combination with gonadotropin-releasing hormone agonists or antagonists confirmed better outcomes of IVF procedures when gonadotropin-releasing hormone agonists were used. There were fewer terminations of procedures (10 vs 20%), higher implantation rates (25.3 vs 10.7%), and higher live birth rates (27.6 vs 13%). These results were valid for young patients with POSEIDON group 3, but they did not confirm any differences when gonadotropin-releasing hormone agonists or antagonists were used in older patients with POSEIDON group 4.(21)

We also analyzed and published our own data on the outcomes of IVF procedures in patients with POR in 2016. We compared 142 IVF procedures (gonadotropin-releasing hormone agonist group), 53 IVF procedures (gonadotropin-releasing hormone antagonist group) and 36 IVF procedures in spontaneous cycles. The average number of oocytes (2.8±1.8) and embryos (1.6±1.2) per aspiration was statistically significantly higher in the agonist group compared to the gonadotropin-releasing hormone antagonist group and spontaneous cycles. The proportion of immature oocytes, fertilization rate and proportion of good quality embryos did not differ between the groups. The pregnancy rate also did not differ statistically significantly (18.9% after gonadotropin-releasing hormone agonists, 10.6% after gonadotropin-releasing hormone antagonists and 5.6% after spontaneous cycles). However, the live birth rate per follicle aspiration was statistically significantly higher in the gonadotropin-releasing hormone agonist group compared to the gonadotropin-releasing hormone antagonist group (15.1% vs. 4.2%; p=0.024). When using gonadotropin-releasing hormone antagonists, we assume that the lower live birth rate is a consequence of a less receptive endometrium due to its premature maturation, which occurs with prolonged ovarian stimulation with gonadotropins in the follicular phase.(22)

One attempt to obtain more oocytes is to stimulate the ovaries with high doses of gonadotropins, but agreement among decision-makers has not yet been reached and has not been credibly supported in many studies.(23) It is increasingly known that with higher doses, although we obtain some more oocytes and reduce the rate of aborted IVF procedures, we do not increase the cumulative live birth rate (24), because this is also significantly influenced by the patient's age, the genetic quality of the oocytes (25), the characteristics of the sperm and the implantation capacity of the endometrium.(26,27) Numerous studies confirm that oocytes obtained by increasing doses of gonadotropins are of poorer quality due to greater nuclear immaturity. This is supported by the fact that there is a certain hierarchy among antral follicles and their ability to respond to gonadotropins with the highest quality oocytes .(23) This line of reasoning leads to the decision to continuously stimulate the ovaries in order to obtain a cohort of the most promising follicles with high-quality oocytes throughout the menstrual cycle. In a study by Ubaldi et al., where they stimulated the ovaries of patients with POR in both the follicular and luteal phases of the same menstrual cycle, they obtained the same number of euploid blastocysts in both phases after injection of MII oocytes. Additional stimulation in the luteal phase thus significantly contributed to a higher number of embryo transfers and a very high birth rate - 66% per euploid blastocyst transfer.(28)

During the first wave of the COVID epidemic, for practical reasons, we were forced to perform continuous ovarian stimulation in thirteen elderly and oncology patients. We froze all embryos, as we did not yet have data on how COVID infection affects embryos and especially pregnancy. Data analysis showed that we obtained a slightly higher number of oocytes and embryos in the luteal phase compared to the follicular phase, but the difference did not reach statistical significance due to the small number of patients included.

The next set of studies are those that confirm an increased cumulative live birth rate after repeated IVF procedures in patients with POR. A large Dutch study confirmed an overall cumulative live birth rate of 56% in 551 patients with POI and the transfer of 1128 fresh and 329 thawed embryos over an 18-month period. Again, the age of the patients was shown to have the most negative impact on the outcome of IVF procedures. In older patients with low ovarian reserve, the cumulative live birth rate was 39%, while in younger patients it was between 65 and 68%, which is the same result as in patients with normal response to ovarian stimulation. (29)

We also analyzed and published data from 395 patients who underwent 810 IVF procedures. The first procedure was performed in 2006, and the patients were then followed up until 2012. The analysis included all repeated IVF procedures and the transfer of all thawed embryos during this period. The cumulative pregnancy and birth rates were calculated according to the age of the patients and IVF. When comparing women with IVF aged less than 38 years and older than 38 years, the cumulative live birth rate was statistically significantly different (50% vs 19%, p= 0.003).(19)

Given that endometrial receptivity and its contribution to successful implantation could be studied separately, it is important to know the euploidy status of the transferred embryos.

The purpose of PGT-A procedures is to determine which embryos have a normal genetic status in terms of chromosome number, as these abnormalities (aneuploidies) most often lead to spontaneous abortions. Currently, an invasive approach is used with a biopsy of the embryo's trophectoderm. Despite its good intentions, this procedure has some drawbacks and currently has no proven positive effect on an unselected patient population. (30) The main problem is the reliability of the results in terms of the representativeness of the biopsy sample for the entire embryo. The greatest challenge is mosaicism, which occurs due to errors during mitosis and means the presence of genetically different cells in the embryo. (31) These cells can be randomly classified as normal cells. However, it is not yet completely clear what the normal level of mosaicism is. In the past, a large number of embryos diagnosed as abnormal-aneuploid were discarded, but recent data suggest that this is probably not the case.(32) Another disadvantage of determining the chromosomal status of an embryo from trophectoderm cells is the invasiveness of the procedure for obtaining these cells, as they are obtained by biopsy, which can damage the embryo.(33) Therefore, non-invasiveness of the method for obtaining genetic material is of utmost importance. One such method is the analysis of the culture medium of cultured embryos. For a long time, such an approach was not possible, as there is relatively little cell-free embryonic DNA in the culture medium, and the analysis methods have limitations in detecting DNA.(34,35) With new approaches, these problems no longer exist (36), and research confirms that the results of this type of genetic testing for the presence of aneuploidies can be fully comparable to the results of a classic blastocyst biopsy .(35) It has even been assumed that by analyzing cell-free DNA from the culture medium of a cultured embryo, it is possible to more representatively determine the degree of mosaicism for the entire embryo, compared to a classic biopsy.(37)

**Selected literature:**

1. Oudendijk JF, Yarde F, Eijkemans MJ, Broekmans FJ, Broer SL. Hum Repro d Update. 2012 Jan-Feb;18(1):1-11 1.

2. Ubaldi F, Vaiarelli A, D' Anna R, Rienzi L. Management of poor responders in IVF: is there anything new? Biomed Res Int 2014:352098

3. Blumenfeld Z. [What Is the Best Regimen for Ovarian Stimulation of Poor Responders in ART/IVF?](https://pubmed.ncbi.nlm.nih.gov/32362870/) Front Endocrinol 2020 Apr 17;11:192

4. Polyzos NP, Devroey P. A systematic review of randomized trials for the treatment of poor ovarian responders: is there any light at the end of the tunnel? Fertil Steril 2011;96:1058–61.

5. Poseidon Group (Patient-Oriented Strategies Encompassing IndividualizeD Oocyte Number), Alviggi C, Andersen CY, Buehler K, Conforti A, De Placido G, et al. A new more detailed stratification of low responders to ovarian stimulation: from a poor ovarian response to a low prognosis concept. Fertil Steril 2016;105:1452–3.

6. Ata B, Kaplan B, Danzer H, Glassner M, Opsahl M, Tan SL, et al. Array CGH analysis shows that aneuploidy is not related to the number of embryos generated. Reprod Biomed Online 2012; 24:614–20.

7. Patrizio P, Vaiarelli A, Levi Setti PE, Tobler KJ, Shoham G, Leong M, et al. How to define, diagnose and treat poor responders? Responses from a worldwide survey of IVF clinics. Reprod Biomed Online 2015;30:581–92.

8. Iliodromiti S, Anderson RA, Nelson SM. Technical and performance characteristics of anti-Mullerian hormone and antral follicle count as biomarkers of ovarian response. Hum Reprod Update 2015;21:698–710.

9. Busnelli A, Papaleo E, Del Prato D, La Vecchia I, Iachini E, Paffoni A, et al. A retrospective evaluation of prognosis and cost-effectiveness of IVF in poor responders according to the Bologna criteria. Hum Reprod 2015;30:315–22.

10. Baerwald AR, Adams GP, Pierson RA. Ovarian antral folliculogenesis during the human menstrual cycle: a review. Hum Reprod Update 2012;18:73–91.

11. Kuang Y, Chen Q, Hong Q, Lyu Q, Ai A, Fu Y, et al. Double stimulations during the follicular and luteal phases of poor responders in IVF/ICSI programs (Shanghai protocol). Reprod Biomed Online 2014;29:684–91.

12. Shapiro BS, Daneshmand ST, Garner FC, Aguirre M, Hudson C. Clinical rationale for cryopreservation of entire embryo cohorts in lieu of fresh transfer. Fertil Steril 2014;102:3 –9.

13. Wang A , Santistevan A , Hunter Cohn K , Copperman A , Nulsen J , Miller BT , Widra E , Westphal LM , Yurttas Beim P. Freeze-only versus fresh embryo transfer in a multicenter matched cohort study: contribution of progesterone and maternal age to success rates. Fertil Steril 2017 Aug;108(2):254-261.

14. Shapiro BS, Daneshmand ST, Garner FC, Aguirre M, Hudson C, Thomas S. Evidence of impaired endometrial receptivity after ovarian stimulation for in vitro fertilization: a prospective randomized trial comparing fresh and frozen-thawed embryo transfer in normal responders. Fertil Steril 2011; 96: 344–348.

15. Horcajadas JA, Riesewijk A, Polman J, van Os R, Pellicer A, Mosselman S, et al. Effect of controlled ovarian hyperstimulation in IVF on endometrial gene expression profiles. Mol Hum Reprod 2005;11:195–205.

16. Huang R, Fang C, Xu S, Yi Y, Liang X. Premature progesterone rise negatively correlated with live birth rate in IVF cycles with GnRH agonist: an analysis of 2,566 cycles. Fertil Steril 2012;98:664–70.

17. Kang HJ, Melnick AP, Stewart JD, Xu K, Rosenwaks Z. Preimplantation genetic screening: who benefits?. Fertile Sterile. 2016;106(3):597-602.

18. Greco, E., Greco, A., & Minasi, MG (2019). Reassuring data concerning follow-up data of children born after preimplantation genetic diagnosis. Fertility and sterility, 111(6), 1111–1112.

19. Vrtačnik U, Vrtačnik Bokal E, Devjak R.Cumulative Delivery Rate after Providing Full Reimbursement In Vitro Fertilization Programme: A 6-Years Survey. Biomed Res Int 2014

20. Leijdekkers JA, Eijkemans MJC, van Tilborg TC, Oudshoorn SC, van Golde RJT, Hoek A, Lambalk CB, de Bruin JP, Fleischer K, Mochtar MH, Kuchenbecker WKH, Laven JSE, Mol BWJ, Torrance HL, Broekmans FJM; OPTIMIST study group. [Cumulative live birth rates in low-prognosis women.](https://pubmed.ncbi.nlm.nih.gov/31125412/) Hum Reprod . 2019 Jun 4;34(6):1030-1041.

21. Huang MC , Tzeng SL , Lee CI, Chen HH , Huang CC, Lee TH , Lee MS . GnRH agonist long protocol versus GnRH antagonist protocol for various aged patients with diminished ovarian reserve: A retrospective study PLoS ONE 2018 Nov 7;13(11):e0207081

22. Stimpfel M, Vrta čnik-Bokal E, Pozlep B, Kmecl J, Virant-Klun I. Gonadotrophin-releasing hormone agonist protocol of controlled ovarian hyperstimulation as an efficient treatment in Bologna-defined poor ovarian responders. Syst Biol Reprod Med. 2016 Aug;62(4):290-6.

23. Jori A Leijdekkers, Helen L Torrance , Nienke E Schouten , Theodora C van Tilborg, Simone C Oudshoorn , Ben Willem J Mol , Marinus JC Eijkemans , [Frank JM Broekmans](https://pubmed.ncbi.nlm.nih.gov/?term=Broekmans+FJM&cauthor_id=31838515) . Individualized ovarian stimulation in IVF/ICSI treatment: it is time to stop using high FSH doses in predicted low responders. Hum Reprod 2020 Sep 1;35(9):1954-1963.

24. Van Tilborg TC, Torrance HL, Oudshoorn SC, Eijkemans MJC, Koks CAM, Verhoeve HR, Nap AW, Scheffer GJ, Manger AP, Schoot BC, Sluijmer AV, Verhoeff A, Groen H, Laven JSE, Mol BWJ, Broekmans FJM . OPTIMIST study group. [Individualized versus standard FSH dosing in women starting IVF/ICSI: an RCT. Part 1: The predicted poor responder.](https://pubmed.ncbi.nlm.nih.gov/29121326/) Hum Reprod. 2017 Dec 1;32(12):2496-2505 .

25. Broekmans FJ, Knauff EA, te Velde ER, Macklon NS, Fauser BC. [Female reproductive ageing: current knowledge and future trends.](https://pubmed.ncbi.nlm.nih.gov/17275321/) Trends Endocrinol Metab. 2007 Mar;18(2):58-65.

26. Simon L, Murphy K, Shamsi MB, Liu L, Emery B, Aston KI, Hotaling J, Carrell DT. [Paternal influence of sperm DNA integrity on early embryonic development.](https://pubmed.ncbi.nlm.nih.gov/25205757/) Hum Reprod . 2014 Nov;29(11):2402-12.

27. Liu KE, Hartman M, Hartman A, Luo ZC, Mahutte N. The impact of a thin endometrial lining on fresh and frozen-thaw IVF outcomes: an analysis of over 40,000 embryo transfers. Hum Reprod 2018:33; 1883-1888.

28. Ubaldi FM, Capalbo A, Vaiarelli A, Cimadomo D, Colamaria S, Alviggi C, Trabucco E, Venturella R, Vajta G, Rienzi L. [Follicular versus luteal phase ovarian stimulation during the same menstrual cycle (DuoStim) in a reduced ovarian reserve population results in a similar euploid blastocyst formation rate: new insight in ovarian reserve exploitation.](https://pubmed.ncbi.nlm.nih.gov/27020168/) Fertile Sterile. 2016 Jun;105(6):1488-1495.

29. Leijdekkers JA, Eijkemans MJC, van Tilborg TC, Oudshoorn SC, van Golde RJT, Hoek A, Lambalk CB, de Bruin JP, Fleischer K, Mochtar MH, Kuchenbecker WKH, Laven JSE, Mol BWJ, Torrance HL, Broekmans FJM; OPTIMIST study group. [Cumulative live birth rates in low-prognosis women.](https://pubmed.ncbi.nlm.nih.gov/31125412/) Hum Reprod . 2019 Jun 4;34(6):1030-1041

30. Munné S, Kaplan B, Frattarelli JL, et al. Preimplantation genetic testing for aneuploidy versus morphology as selection criteria for single frozen-thawed embryo transfer in good-prognosis patients: a multicenter randomized clinical trial. Fertile Sterile. 2019;112(6):1071-1079.e7. doi:10.1016/j.fertnstert.2019.07.1346

31. Popovic M, Dhaenens L, Boel A, Menten B, Heindryckx B. Chromosomal mosaicism in human blastocysts: the ultimate diagnostic dilemma [published correction appears in Hum Reprod Update. 2020 Apr 15;26(3):450-451]. Hum Reprod Update. 2020;26(3):313-334. doi:10.1093/humupd/dmz050

32. Gleicher N, Kushnir VA, Barad DH. How PGS/PGT-A laboratories succeeded in losing all credibility. Reprod Biomed Online. 2018;37(2):242-245. doi:10.1016/j.rbmo.2018.06.019

33. Leaver M, Wells D. Non-invasive preimplantation genetic testing (niPGT): the next revolution in reproductive genetics? Hum Reprod Update. 2020;26(1):16-42. doi:10.1093/humupd/dmz033

34. Shamonki MI, Jin H, Haimowitz Z, Liu L. Proof of concept: preimplantation genetic screening without embryo biopsy through analysis of cell-free DNA in spent embryo culture media. Fertile Sterile. 2016;106(6):1312-1318. doi:10.1016/j.fertnstert.2016.07.1112

35. Kuznyetsov V, Madjunkova S, Abramov R, et al. Minimally Invasive Cell-Free Human Embryo Aneuploidy Testing (miPGT-A) Utilizing Combined Spent Embryo Culture Medium and Blastocoel Fluid -Towards Development of a Clinical Assay. Sci Rep. 2020;10(1):7244. Published 2020 Apr 29. doi:10.1038/s41598-020-64335-3

36. Babariya D. Non-invasive preimplantation genetic testing for aneuploidy (NI PGT-A). Reprod Biomed Online. 2019;38(Suppl 1):e10-e11. doi.org/10.1016/j.rbmo.2019.03.020

37. Huang L, Bogale B, Tang Y, Lu S, Xie XS, Racowsky C. Noninvasive preimplantation genetic testing for aneuploidy in spent medium may be more reliable than trophectoderm biopsy. Proc Natl Acad Sci US A. 2019;116(28):14105-14112. doi:10.1073/pnas.1907472116

38. Chandramouli K, Qian PY. Proteomics: challenges, techniques and possibilities to overcome biological sample complexity. Hum Genomics Proteomics. 2009;2009:239204. Published 2009 Dec 8. doi:10.4061/2009/239204

39. Janša V , Klančič T, Vrtačnik Bokal E, Ban Frangež H, Lanišnik Rižner T. Proteomic analyses of peritoneal fluid cartilage oligomeric matrix protein and transforming growth factor-beta-induced protein ig-h3 as new candidate biomarkers for endometriosis . The article is under revision.

40. Abdulkhalikova D, Jančar N, Jensterle M, Šuštaršič A, Bokal Vrtačnik E. The influence of lifestyle modifications on anthropometric, metabolic and reproductive outcomes and endometrial proteome of women with polycystic ovarian syndrome and obesity. The article is under revision.

3. Purpose of the research

- We predict that by continuously stimulating the ovaries (both in the follicular and luteal phases) we will obtain a greater number of oocytes in one procedure than in the previous two procedures combined, where we performed ovarian stimulation only in the follicular phase. At the same time, we assume that with a larger cohort of stimulated follicles we will also have a higher probability of obtaining higher-quality oocytes, which will lead to a greater number of good-quality embryos. Freezing all embryos will allow us to prolong ovarian stimulation with gonadotropins, because we will not avoid the increase in progesterone and the resulting premature maturation of the endometrium, which negatively affects embryo implantation.
- Women who require repeated IVF procedures often drop out of treatment due to psychological stress, so continuous stimulation would represent a smaller and shorter burden on patients. Usually, 3 months pass between the completed and new procedure, but with continuous stimulation, the procedure would be completed within 1 month, which is especially important for older patients.
- The next goal is to differentiate between euploid and aneuploid embryos based on non-invasive DNA analysis from the culture medium. This would avoid potential damage to the embryo after the ectoderm biopsy required for PGT.
- We anticipate that we will be able to determine the protein profile characteristic of endometrial receptivity by comparing the proteomes of spontaneous and stimulated cycles, and by comparing the proteomes of those women who conceived and those who did not. This could confirm the clinical predictions that freezing and then transferring embryos in a spontaneous cycle is a more appropriate option for patients with POR.

4. People who will be invited to participate in the study

The proposed study will prospectively include all consecutive women with POR on gonadotropin stimulation before the age of 43 undergoing IVF procedures at Department of Human Reproduction, Division of Obstetrics and Gynaecology, University Medical Centre Ljubljana, who have not achieved pregnancy or birth after two IVF procedures. We plan to include 100 patients over a two-year period. We will exclude all women who are treated with IVF for a severe male cause of infertility.

Upon inclusion in the study, the couples will be adequately informed about the plan and purpose of the study. All women will sign a written consent form for participation in the study (attached). We declare that consent to participate in the study will be a free choice of the subject and that the invitation to participate will not be accompanied by pressure or inappropriate inducement. Patients who do not wish to participate in the study will receive the same treatment as usual.

5. Methods

Although a single definition of POR has not yet been adopted, the most commonly used is the Bologna classification. This defines POR by patient age, poor response to ovarian stimulation in previous procedures, reduced antral follicle count (AFC), and reduced serum anti-Mullerian hormone (AMH) concentration.

In the proposed study, patients will be classified according to the latest POSEIDON classification (Patient Oriented Strategies Encompassing Individualized Oocyte Number) (5), which takes into account the patient's age, the expected degree of aneuploidies in embryos, markers of ovarian reserve (AMH, AFC), and the response to ovarian stimulation in the previous IVF procedure.

We will divide the patients into 4 groups:

POSEIDON group 1: Patients younger than 35 years with normal ovarian reserve markers (AMH>1.2ng/mL, AFC >5) and unexpected POR (subgroups: 1a: <4 retrieved oocytes (RO); 1b: 4-9 retrieved RO).

POSEIDON group 2: Patients older than 35 years with normal markers of ovarian reserve (AMH>1.2ng/mL, AFC>5) and unexpected POR (subgroups: 1a: <4 RO; 1b: 4-9 RO).

POSEIDON group 3: Patients younger than 35 years with POR (AMH<1.2ng/mL, AFC<5).

POSEIDON group 4: Patients older than 35 years with POR (AMH<1.2ng/mL, AFC<5).

We will introduce a uniform protocol for ovarian stimulation in the follicular and luteal phases of the menstrual cycle in all patients. On the twenty-first day of the cycle, 4 mg of estradiol will be introduced to synchronize and coordinate follicle growth.(10) On day 2 of the next menstrual cycle, we will discontinue estradiol and begin ovarian stimulation with gonadotropins (rFSH 300 IU/day). Gonadotropin-releasing hormone antagonists will be started on day 7 of the cycle, both in the follicular and luteal phases. A GnRH agonist (Gonapeptyl 0.1 mg sc) will be used to mature the oocytes, when the follicles reach a size of 17-18 mm in diameter. Follicle aspiration will be performed 36 hours after the administration of the agonist. Five days after the first follicle aspiration, when complete luteolysis has occurred, stimulation will begin in the same way as in the follicular phase. Fertilization, culture to blastocyst, and vitrification will be performed according to established laboratory methods. After thawing the embryos, the blastocysts will be transferred in the next spontaneous or stimulated cycle. The culture media in which the embryos will be grown will be stored at -80°C for later non-invasive PGT-A. The new method will be developed and first validated in a clinical PGT programme. In this way, we will be able to separately determine the influence of the endometrium or embryo quality on the pregnancy rate after IVF procedures in patients with POR.

The non-invasive method of determining the chromosomal status of the embryo after isolating DNA from the culture medium in which the embryo will be cultured will first be validated in embryos in the regular clinical PGT program. Only then will we use the non-invasive approach to analyze the chromosomal status of embryos in our target population of patients with POR. More precisely, this means that the embryos in the PGT-SR program will be cultured in the same way as they are cultured in clinical practice (day 3 of preimplantation development, individual culture in drops with a volume of 40 microliters). On this day, an opening will be made in the zona pellucida of the embryo with a laser - this way the blastocyst can be more easily separated and therefore it is also easier to biopsie a fragment of the trophoblast for genetic analysis. For comparison with the invasive procedure, 10 microliters of the culture medium in which the embryo is cultured will be taken just before the biopsy and then genetically tested. After confirming the reliability of the results, we will use the same method in our target population of patients with POR. The embryos of these patients will be cultured in exactly the same way as usual. The only difference will be that from day 3 onwards, these embryos will be cultured individually in drops of culture medium. On day 3, the embryos will also be thoroughly washed with fresh culture medium in order to remove any remaining DNA from granulosa cells or sperm from their surface, which could give distorted data in the analysis.

We assume that in patients with POR, the low pregnancy rate is influenced by both the reduced number of obtained MII cells with a higher number of aneuploid embryos, as well as the altered receptivity of the endometrium, therefore, to assess the receptivity of the endometrium, we will determine the protein expression profile during the expected implantation window.

Due to the high cost, we will not be able to perform an endometrial profile in all patients. We anticipate that we will be able to determine the implantation window based on inferences from the analysis of the protein profile in 30 patients. From POSEIDON group 1, we will perform an endometrial biopsy in five patients from subgroup 1a and five patients from subgroup 1b. We will also perform an endometrial biopsy in the same way from patients from group 2. From groups 3 and 4, we will perform an endometrial biopsy in five women from each group. In each patient, we will analyze the proteins with microarrays 2 times, namely in a spontaneous and a stimulated cycle.

To assess the protein expression profile in spontaneous menstrual cycles, a first endometrial biopsy will be performed in all subjects during the implantation window period (days 20–24). The exact day of ovulation will be determined by ovulation tests. Samples will be collected with a plastic pipet (Rampipella Ri.Mos.SRL Mirandola, Italy), snap-frozen and stored at -80 °C until final analysis.

The second endometrial biopsy will be performed five days after the first egg aspiration (LH+7).

During an endometrial biopsy, a piece of the mucosa will also be sent for histopathological examination to rule out any chronic inflammation in the uterine cavity, which could negatively affect the outcome of the procedures.

When performing continuous stimulation, we will obtain information on how the protein profile in the endometrium of the implantation window of a spontaneous menstrual cycle differs from the protein profile in the endometrium after stimulation with high doses of gonadotropins, which may subsequently affect embryo implantation and development. Protein profiling will be performed using protein arrays, which enable high-throughput targeted studies of a larger set of proteins in different biological samples (38). This new technology is highly suitable for the search for new biochemical markers . In our previous study, we used a protein array (Sciomics GmbH, Heidelberg, Germany), which can identify 1438 different proteins with 1925 antibodies and has proven to be suitable for endometrial protein profiling, and we will therefore also send the samples to the aforementioned laboratory (39,40).

Using a non-invasive method of determining the chromosomal status of the embryo after DNA isolation from the culture medium (PGT-A), in which the embryo will be cultured, we will try to establish a new method of determining euploid blastocysts in collaboration with geneticists. The method is also still being developed and tested worldwide. The advantage of the proposed method compared to trophectoderm biopsy is that it avoids possible damage to the embryo. Blastocyst selection would be based on the analysis of amplified DNA in the culture medium.

The primary outcomes of the research will be:

- Number of eggs
- Number of MII oocytes
- Fertilization rate
- Number of embryos
- Number of high quality embryos
- Pregnancy rate

All of the above outcomes (1-6) will be evaluated by comparing the results of continuous stimulation with the previous two IVF procedures together.

- Difference in endometrial protein profile between spontaneous and stimulated cycles
- Difference in endometrial protein profile between women who will conceive and those who will not.

Secondary research outcomes:

- Proportion of aneuploid embryos in patients with POR under 35 years of age
- Proportion of aneuploid embryos in patients with POR older than 35 years
- Average gonadotropin consumption per egg cell in the previous two IVF procedures compared to the continuous procedure.

Statistical data processing:

We will use descriptive statistics methods. From statistical tests, we will use logistic regression methods, Mann Whitney test and, if necessary, Fisher's exact test. The statistical significance of the correlation between marker concentrations will be determined by Spearman Rank correlation analysis. The statistical significance of the results will be discussed at p <0.05. SPSS version 19.0 software will be used for analysis, and other software if necessary.

6. Summary of the research plan

In the treatment of infertility with IVF procedures, we sometimes encounter a problem related to poor ovarian response (POR) to stimulation. The prognosis of patients with POR largely depends on the age of the patient and the number of oocytes retrieved. Data from the literature show that reduced ovarian response occurs in 9-24% of infertile women . The prevalence increases especially with the postponement of pregnancy to the late thirties or even forties. In approximately half of these patients, the cause is unknown. In the desire to increase the live birth rate, numerous ovarian stimulation protocols are used.

In fact, the number of oocytes retrieved and the age of the woman have the greatest impact on the success of conception. Therefore, predicting ovarian response by individually planning stimulation is very important, especially in patients with reduced ovarian response. At present, there is insufficient evidence to support the superiority of any of the various known stimulation protocols in women with reduced ovarian response. It is only known that the live birth rate is lower in them compared to infertile women with normal or increased ovarian response to stimulation.

Recently, scientists have come to the realization that more follicles are recruited in one menstrual cycle (including the second half) and in more waves than previously thought. This discovery led to the idea that by double stimulation of the ovaries – both in the first and second half of the menstrual cycle – a larger number of eggs and embryos could be obtained in one menstrual cycle. The embryos would then be frozen and transferred in subsequent cycles, as we know that the pregnancy rate with fresh or thawed embryos is comparable. The first experience with double stimulation was described by Kuang and colleagues, who reported the same number and developmental potential of the retrieved eggs in the first and second phases of the cycle. During the first wave of the COVID-19 epidemic, we also did this at the our clinic in older women and obtained even a few more cells in the second phase compared to the first.

Given that successful fertilization requires the synchronous interaction of a receptive endometrium and high-quality embryos with a normal chromosome number, current research is increasingly focused on analyzing the protein profile of the endometrium with the aim of identifying and determining the optimal time for embryo implantation. It is assumed that exposure to ovarian stimulation with drugs and the resulting altered hormone levels impairs the receptivity of the endometrium to the embryo (receptivity), which reduces the pregnancy rate, especially with poorer quality embryos. There is increasing evidence that the endometrium in thawed embryo transfer cycles (i.e. without ovarian stimulation) is more receptive compared to the endometrium after ovarian stimulation. Hormonal regulation plays an important role in the receptivity of the endometrium, and high concentrations of estrogen and progesterone may affect the expression of genes and proteins involved in the embryo implantation process. Asynchrony between the embryo and the non-receptive uterine lining may be the result of ovarian stimulation, which causes a premature increase in progesterone and consequently premature maturation of the lining, which disrupts the implantation window period, which is the optimal time for embryo implantation.

The Act on Infertility Treatment and Assisted Reproductive Technology (Article 31) stipulates that genetic testing of an early embryo may only be performed in cases of risk of a serious hereditary disease or if this is necessary for the success of the biomedically assisted fertilization procedure. In Slovenia, these procedures have been performed since 2004, and only in cases of risk of transmitting a serious hereditary disease to the offspring. We do not perform such tests with the aim of improving the success of IVF procedures, as it is not yet completely clear on a global scale in which cases this would make sense. For the time being, it seems that they are particularly suitable for older women, as this can reduce the incidence of miscarriage and shorten the time to conception. Preimplantation genetic testing (PGT) procedures, which are performed with the aim of preventing the birth of a child with a serious hereditary disease, can be further divided into PGT-M (monogenic defects), in which embryos are tested to prevent the transmission of monogenic diseases, and PGT-SR (structural rearrangements), in which embryos are examined to prevent the transmission of structural chromosomal abnormalities. The method of examination that attempts to improve the success of IVF procedures is called PGT-A (aneuploidy); in this method, embryos are examined for the number of chromosomes or the presence of aneuploidy .

For genetic examination of the embryo, it is necessary to biopsy it. The biopsy can be performed at the stage of the low-cell embryo on the third day of development or at the blastocyst stage on the 5/6th day of development. The embryo on the third day of development normally has 6-8 cells, which represents a limitation in the amount of DNA. When biopsying at this stage of development, 1 or a maximum of 2 cells (blastomeres) are removed from the embryo. Research shows that removing 1-2 cells can hypothetically negatively affect the further development and implantation potential of the embryo. Another disadvantage of this approach is that it is not possible to reliably determine mitotic errors that lead to mosaicism. The advantage of the procedure is that it allows the separation of meiotic errors or mutations from both parents.

An approach that provides more reliable results is an embryo biopsy at the blastocyst stage. At this stage, the embryo usually has around 100 cells, and the part of the embryo that develops into the fetus (embryoblast) and the part of the embryo that develops into the placenta (trophoblast) can already be distinguished. The biopsy is performed by cutting off a small part of the trophoblast ( around 5-10 cells) with the help of a laser , and the embryo is then frozen in liquid nitrogen for the duration of the genetic examination . Another advantage is that only embryos with the potential for implantation are biopsied. The disadvantage of this method is that mosaicism may occur in a certain proportion, which is less problematic than in the case of a biopsy of low-cell embryos. Although research results show that PGT procedures do not have a negative impact on the health and development of children, due to the relatively invasive intervention on the embryo, concerns constantly arise that such procedures could negatively affect the health of children.

, less invasive and non-invasive procedures for obtaining genetic material from the embryo are constantly being sought , as they could avoid all concerns about direct intervention in it. A less invasive procedure than a blastocyst biopsy is a blastocoel biopsy, which involves aspirating fluid from the blastocyst cavity (blastocoel), where the embryo's DNA is also located, and the blastocyst collapses during this procedure. This procedure is sometimes even used in clinical practice before freezing embryos, as it is said to improve the survival of the blastocyst, although it does involve some intervention in the embryo. A completely non-invasive procedure is genetic analysis of the culture medium in which the embryos are grown . This is possible because embryos secrete parts of nuclear and mitochondrial DNA during development, which can be detected with modern molecular methods and then determined the genetic status of the embryo in terms of the correct number of chromosomes. An abnormal number of chromosomes, even if the embryo successfully implants , usually leads to miscarriage, which exposes the patient to unnecessary stress and also prolongs the time until a possible new pregnancy. All of the above is very unfavorable for patients in the late reproductive period or with impending premature ovarian failure.

We are planning a clinical study with the aim of optimizing the IVF procedures for patients with known POR. Based on data from the literature, we will perform a procedure with double ovarian stimulation in this group of patients - both in the follicular and luteal phases of the cycle. At the same time, we will try to assess the receptivity of the uterine mucosa in natural menstrual cycles and after ovarian stimulation and thus determine the proteins that play a key role in determining the optimal time for embryo implantation. In addition, we will try to introduce a non-invasive method for determining the chromosomal status of the embryo by analyzing the culture medium in which the embryos were grown.

The proposed study will prospectively include all consecutive women with POR to gonadotropin stimulation, undergoing IVF procedures at the Department of Human Reproduction, Division of Obstetrics and Gynaecology, University Medical Centre Ljubljana, who have not achieved pregnancy and birth after two procedures. It is planned to include 100 patients over a two-year period.

In the proposed study, we will classify patients according to the latest POSEIDON classification (Patient Oriented Strategies Encompassing Individualized Oocyte Number), which takes into account the patient's age, the expected degree of chromosome number deviation in embryos, markers of ovarian reserve (antral follicle count (AFC), anti-Mullerian hormone (AMH) concentration), and the response to ovarian stimulation in a previous in IVF procedures.

We will divide the patients into 4 groups:

POSEIDON group 1: Patients younger than 35 years with normal ovarian reserve markers (AMH>1.2ng/mL, AFC >5) and unexpected POR (subgroups: 1a: <4 retrieved oocytes (RO); 1b: 4-9 retrieved RO).

POSEIDON group 2: Patients older than 35 years with normal markers of ovarian reserve (AMH>1.2ng/mL, AFC>5) and unexpected POR (subgroups: 1a: <4 RO; 1b: 4-9 RO).

POSEIDON group 3: Patients younger than 35 years with POR (AMH<1.2ng/mL, AFC<5).

POSEIDON group 4: Patients older than 35 years with POR (AMH<1.2ng/mL, AFC<5).

We will introduce a uniform protocol with ovarian stimulation in the follicular and luteal phases of the menstrual cycle in all patients. On the twenty-first day of the cycle, we will introduce 4 mg of estradiol valerate to synchronize and coordinate follicle growth. On day 2 of the menstrual cycle, we will discontinue estradiol valerate and begin ovarian stimulation with gonadotropins (rFSH 300 IU/day). Gonadotropin-releasing hormone antagonists will be started on day 7 of the cycle, both in the follicular and luteal phases. A GnRH agonist (Gonapeptyl 0.1 mg sc) will be used to mature the oocytes, when the follicles reach a size of 17-18 mm in diameter. Follicle aspiration will be performed 36 hours after the administration of the GnRH agonist. Five days after the first follicle aspiration, stimulation will begin in the same way as in the follicular phase. Fertilization, culture to blastocyst and freezing of embryos will be performed according to established laboratory methods. After thawing the embryos, the blastocysts will be transferred in the next spontaneous or stimulated cycle. The culture media in which the embryos will be grown will be stored at -80°C for later non-invasive PGT-A. The new method will be developed and first validated in a clinical PGT programme. In this way, we will be able to separately determine the influence of the uterine mucosa or embryo quality on the pregnancy rate after in vitro fertilization procedures in patients with reduced ovarian response.

The non-invasive method of determining the chromosomal status of the embryo after isolating DNA from the culture medium in which the embryo will be cultured will first be validated in embryos in the regular clinical PGT program. Only then will we use a non-invasive approach to analyze the chromosomal status of embryos in our target population of patients with reduced ovarian response. More precisely, this means that we will culture embryos in the PGT-SR program in the same way as we otherwise culture them in clinical practice (on the 3rd day of preimplantation development, individual culture in drops with a volume of 40 microliters). On this day, we will make an opening in the embryo envelope with a laser - this way the blastocyst can be more easily separated and therefore it is also easier to biopsie a fragment of the trophoblast for genetic analysis. For comparison with the invasive procedure, we will take 10 microliters of the culture medium in which the embryo is cultured just before the biopsy and then genetically test it. After confirming the reliability of the results, we will use the same method in our target population of patients. The embryos of these patients will be cultured in exactly the same way as usual. The only difference will be that from day 3 onwards, these embryos will be cultured individually in drops of culture medium. On day 3, the embryos will also be thoroughly washed with fresh culture medium in order to remove any remaining DNA from granulosa cells or sperm from their surface, which could give distorted data in the analysis.

We assume that in patients with reduced ovarian reserve, the low pregnancy rate is influenced by both the reduced number of retrieved mature oocytes with a higher number of embryos with an abnormal number of chromosomes, as well as the altered receptivity of the uterine mucosa, therefore, to assess receptivity, we will determine the protein expression profile in the uterine mucosa during the expected implantation window. In women with double ovarian stimulation, an endometrial biopsy and uterine cavity lavage will be taken before entering the IVF procedure, during the implantation window period, and the same procedure will be repeated 5 days after the first follicle aspiration or immediately before the start of stimulation in the luteal phase. This is the time suitable for embryo transfer in fresh in vitro fertilization procedures. All samples will be stored at -80 °C for later simultaneous analysis. When performing double stimulation, we will obtain information on how the protein profile in the endometrium during the implantation window of a spontaneous menstrual cycle differs from the protein profile in the mucosa after stimulation with high doses of gonadotropins, which may subsequently affect implantation and embryo development. The protein profile will be determined using protein arrays that enable high-throughput targeted studies of a larger set of proteins in various biological samples. Due to the high cost, we will not be able to perform the protein profile in the mucosa in all subjects. We anticipate that we will be able to determine the implantation window based on inferences from the analysis of the protein profile in 30 patients. From POSEIDON group 1, we will perform sampling from five patients from subgroup 1a and five patients from subgroup 1b. We will also perform sampling from patients from group 2 in the same way. From groups 3 and 4, we will perform sampling from five women from each group. In each patient, we will analyze proteins using microarrays twice, in a spontaneous and stimulated cycle.

Using a non-invasive method of determining the chromosomal status of the embryo after DNA isolation from the culture medium (PGT-A), in which the embryo will be cultured, we will try to establish a new method of determining the chromosomal status of blastocysts in collaboration with geneticists. The method is also still being developed and tested worldwide. The advantage of the proposed method compared to trophectoderm biopsy is that it avoids possible damage to the embryo. Blastocyst selection would be based on the analysis of amplified DNA in the culture medium.

The primary outcomes of the research will be:

- Number of eggs
- Number of mature oocytes
- Fertilization rate
- Number of embryos
- Number of high quality embryos
- Pregnancy rate

All of the above outcomes (1-6) will be evaluated by comparing the results of continuous stimulation with the previous two IVF procedures together.

- Difference in the protein profile in the endometrium between spontaneous and stimulated cycles
- Difference in the protein profile in the endometrium between women who will become pregnant and those who will not become pregnant.

Secondary research outcomes:

- Proportion of embryos with abnormal chromosome numbers in patients with POR under 35 years of age
- Proportion of embryos with abnormal chromosome numbers in patients with POR over 35 years of age
- Average gonadotropin consumption per egg cell in the previous two IVF procedures compared to the continuous procedure.

7. Assessment of ethical aspects of the research

We believe that the research is ethically sound because we will ensure the following throughout the process:

- The inclusion of patients in the study will be voluntary and even if the subjects refuse to participate, this will not affect the course of their treatment;
- Blood sampling to determine hormone concentration (AMH), antral follicle count (AFC) measurement, ovarian stimulation and follicle aspiration are part of routine and well-established diagnostic and therapeutic procedures;
- Embryo freezing is part of routine IVF procedures and does not pose a major risk to embryo viability;
- Transfer of thawed embryos does not pose a risk of reducing the pregnancy rate, as it has already been proven that the pregnancy rate with fresh or thawed embryos is comparable ;
- Taking a uterine cavity lavage and endometrial biopsy are painless procedures that do not pose any risk to the patient and do not affect the success of infertility treatment procedures;
- Determination of the chromosomal status of the embryo after DNA isolation from the culture medium (PGT-A) is a non-invasive method and does not pose a risk to embryo viability.

8. Will participants have access to their health data and research results?

Subjects who wish to be informed about the results of the study will be able to request in writing a short report, which will be prepared specifically for them at the end of each phase of the study. If they wish to find out the results of their own samples, we will provide them with the results in a suitable format.

9. Safety of research subjects and protection of confidentiality of personal data

Subjects in the study will be treated according to the same, generally accepted principles of infertility treatment. Subjects may withdraw from the study at any time, without giving a reason. Withdrawal from the study will not affect further medical treatment. If a complication is suspected in the subjects (more likely due to established treatment procedures than due to modifications for the purpose of the study), the subjects will be able to contact the responsible person, Prof. Dr. Eda Bokal Vrtačnik, MD, at the telephone number: 01/522-60-60.

Modification of the existing ovarian stimulation protocol for the target population of women with POR presents minimal potential risks to the health of the subjects. The most common complication of IVF is ovarian hyperstimulation syndrome, which is practically impossible in our target population of subjects, because of the proven reduced ovarian responsiveness to stimulation. There may be other risks, similar to all conventional IVF procedures, such as complications due to bleeding or inflammation during the invasive follicle aspiration procedure. All researchers in the study are able to recognize these complications and are able to take appropriate action to treat them. Our department provides both outpatient and inpatient (drug and surgical) treatment of complications.

The protection of personal data of the persons included in the research will be carried out by means of a special coded labeling of the samples, from which the personal data of the subjects will not be visible. The database will be stored by the principal investigator, Prof. Dr. Eda Bokal Vrtačnik, MD, and will not be accessible to others.

The researchers commit to adhering to the principles of the Declaration of Helsinki on Biomedical Research Involving Human Subjects, the Council of Europe Convention for the Protection of Human Rights and Dignity of the Human Being with regard to the Application of Biology and Medicine (Oviedo Convention), and the principles of the Slovenian Code of Medical Ethics.

The study will be conducted on women of childbearing potential, but without an increased risk of teratogenicity, reduced fertility, or danger in the event of a potential pregnancy. In the event that the study identifies important data that would be therapeutically and/or prognostically important for the subjects, the participants in the study will be informed and appropriately instructed.

10. Funding

The research physicians will not receive financial compensation for their participation in the study. Infertility treatment is part of regular clinical treatment and no additional financial resources will be required for this. Additional funds for the analysis of uterine cavity lavage and endometrial biopsies will be provided from the funds for the tertiary project of the University Medical Centre Ljubljana. All additional research procedures will be carried out at the expense of research time and money.

11. Attachments

- Statement by the principal investigator of the Department of Human Reproduction, University Medical Centre Ljubljana
- Statement by the Head of the Department of Human Reproduction, University Medical Centre Ljubljana
- Statement from the Director of the Division of Obstetrics and Gynaecology, University Medical Centre Ljubljana
- Statements from research participants
- Statement by the Head of the Clinical Institute for Genomic Medicine
- Written information about the study for the subjects and the form of declaration of informed and free consent of the participants
- Confirmation of payment of the fee
