# Supplementary material for: Distinct endometrial protein profiles in spontaneous and stimulated cycles in women with poor ovarian response: A prospective case-crossover clinical trial
Source: PLoS One. 2026 May 19;21(5):e0338812. doi: 10.1371/journal.pone.0338812 (PMC13186353; doi:10.1371/journal.pone.0338812)
Supplement: S3 Protocol — (DOCX) [file pone.0338812.s003.docx]

VLOGA ZA MNENJE O ETIČNI SPREJEMLJIVOSTI PREDLOGA RAZISKAVE
 **Naslov raziskave:**

»Kontinuirano spodbujanje jajčnikov z gonadotropini pri ženskah z zmanjšanim odzivom jajčnikov v postopkih zunajtelesne oploditve«.

»Continuous ovarian stimulation with gonadotropins in women with poor ovarian response during in vitro fertilization procedures«.

**Tip raziskave:** prospektivna navzkrižna odprta raziskava

**Odgovorna raziskovalka:** Prof. dr. Eda Bokal Vrtačnik, dr. med., višja svetnica (šifra raziskovalca pri ARRS 12177), specialistka ginekologije in porodništva, predstojnica KO za reprodukcijo Ginekološke klinike Ljubljana.

**Zdravnica, odgovorna za varnost oseb v raziskavi:** Prof. dr. Eda Bokal Vrtačnik, dr. med, višja svetnica, specialistka ginekologije in porodništva

**Predvideno trajanje raziskave:** 3 leta

**Sodelujoči v raziskavi:**

- Prof. dr. Borut Peterlin, dr. med., svetnik (šifra raziskovalca pri ARRS 10458), predstojnik Kliničnega inštituta za genomsko medicino
- Doc. dr. Nina Jančar, dr. med. (šifra raziskovalca pri ARRS 25612), specialistka ginekologije in porodništva, Ginekološka klinika Ljubljana
- Dr. Martin Štimpfel, univ. dipl. bioteh. (šifra raziskovalca pri ARRS 33917), vodja Laboratorija za oploditev z biomedicinsko pomočjo, Ginekološka klinika Ljubljana
- Dzhamilyat Abdulkhalikova, dr.med., specialistka ginekologije in porodništva, Ginekološka klinika Ljubljana

**Ustanove:**

Raziskava je predvidena na Kliničnem oddelku za reprodukcijo, Ginekološka klinika, Univerzitetni Klinični center Ljubljana, kjer bodo potekali klinični pregled, informiran pristanek preiskovank, odvzem izpirka maternice in biopsija endometrija, zdravljenje neplodnosti ter analiza pridobljenih rezultatov. Postopek izventelesne oploditve in nadaljnje gojenje zarodkov, biosija fragmenta trofoblasta, vitrifikacija zarodkov in odvzem gojišča za neinvazivno preiskavo bo potekal v Laboratoriju za oploditev z biomedicinsko pomočjo KO za reprodukcijo GK Ljubljana.

- Neinvazivna metoda določitve kromosomskega statusa zarodka po izolaciji DNK iz gojišča bo potekala na Kliničnem inštitutu za genomsko medicino UKC Ljubljana
- Določitev profila proteinov v izpirkih maternice in bioptatih endometrija bo potekala v laboratoriju Sciomics GmbH, v Heidelberg (Nemčija).

Poln naslov raziskave: **»Kontinuirano spodbujanje jajčnikov z gonadotropini pri ženskah z zmanjšanim odzivom jajčnikov v postopkih zunajtelesne oploditve«.**

1. Znanstvena izhodišča in predstavitev problema

Pri zdravljenju nepolodnosti s postopki zunajtelesne oploditve (ZTO) kljub znanstvenim in tehnološkim napredkom še vedno naletimo na problem, povezan z zmanjšano odzivnostjo jajčnikov (ZOJ). Te pacientke ne predstavljajo homogene skupine in njihova prognoza je v veliki meri odvisna od starosti pacientke in števila pridobljenih jajčnih celic.(1) Podatki iz literature kažejo, da zmanjšan odziv nastopi pri 9-24% neplodnih žensk.(2) Prevalenca se še zlasti povečuje zaradi odmikanja nosečnosti v pozna trideseta leta ali celo štirideseta leta. Pri približno polovici teh pacientk vzrok ni znan.(3) Medtem, ko je zmanjšanje števila antralnih foliklov zaradi staranja dobro znan in pojasnjen pojav (3), mnogo manj vemo o genetski in kromosomski etiologiji ter o metaboličnih, encimskih, iatrogenih, toksičnih, avtoimunih oz vnetnih vzrokih.(3)

Znane so številne definicije za opredelitev žensk z ZOJ. V želji po zvišanju stopnje živorojenih otrok se uporabljajo številni protokoli spodbujanja jajčnikov.(4)

Za natančno opredelitev žensk z ZOJ se v zadnjem času največkrat uporablja klasifikacija POSEIDON (Patient-Oriented Strategies Encompassing Individualized Oocyte Number).(5) Dejstvo je, da število pridobljenih jajčnih celic in starost ženske najbolj vplivata na uspešnost zanositve.(6,7) Torej je napovedovanje odziva jajčnikov z individualnim načrtovanjem spodbujanja zelo pomembno, še zlasti pri pacientkah z ZOJ. Število antralnih foliklov in serumska koncentracija AMH najuspešneje napovedujeta rezervo jajčnikov in posledično tudi odziv na spodbujanje z gonadotropini.(8)

Trenutno nimamo dovolj dokazov, ki bi potrjevali prednost katerega od različnih znanih protokolov spodbujanja pri ženskah z ZOJ. Znano je le to, da je stopnja živorojenih otrok pri njih nižja v primerjavi z neplodnimi ženskami z normalnim ali zvišanim odzivom.(9)

Spoznanje, da rekrutacija kohorte foliklov ne nastopi samo v folikularni fazi, ampak 2 do 3 krat v enem menstruacijskem ciklusu, torej tudi v lutealni fazi (10), vodi v razmišljanje, da bi s kontinuiranim spodbujanjam lahko pridobili večje število jajčnih celic in zarodkov v enem menstruacijskem ciklusu. Zarodke bi nato zamrznili in jih prenesli v naslednjih ciklusih, saj vemo, da je stopnja zanositve s svežimi ali odmrznjenimi zarodki primerljiva. Prvo izkušnjo z dvojnim spodbujanjem je opisal Kuang s sod., ki je poročal o enakem številu in razvojnem potencialu pridobljenih jajčnih celic v folikularni in lutealni fazi.(11) V času prvega vala epidemije COVID-19 smo tako ravnali tudi na Ginekološki kliniki v Ljubljani pri starejših ženskah in pridobili v lutealni fazi celo nekaj več celic v primerjavi s folikularno.

Glede na to, da je za uspešno oploditev potrebno medsebojno sinhrono delovanje receptivnega endometrija in evploidnih zarodkov visoke kakovosti, se trenutne raziskave vedno bolj usmerjajo tudi v analizo profila proteinov v endometriju z namenom prepoznavanja in določitve optimalnega vgnezditvenega okna.

Predvideva se, da izpostavljenost spodbujanju jajčnikov z gonadotropini in posledični spremenjeni ravni hormonov poslabša receptivnost endometrija, kar zmanjša stopnjo zanositve zlasti pri zarodkih slabše kakovosti.(12,13) Vedno več je dokazov, da je endometrij v ciklusih prenosa odmrznjenih zarodkov bolj receptiven v primerjavi z endometrijem po spodbujanju jajčnikov.(14) Hormonska regulacija ima pomembno vlogo pri receptivnosti endometrija, visoka koncentracija estrogena in progesterona pa lahko vpliva na izražanje genov in proteinov, vključenih v proces vgnezditve zarodkov.(15) Asinhronija med zarodkom in nereceptivnim endometrijem je lahko posledica spodbujanja jajčnikov, ki povzroči prezgodnji porast progesterona in posledično prezgodnje dozorevanje endometrija, kar zmoti obdobje vgnezditvenega okna.(16)

Zakon o zdravljenju neplodnosti in postopkih oploditve z biomedicinsko pomočjo v 31. členu narekuje, da se genska preiskava zgodnjega zarodka sme opraviti samo v primeru nevarnosti hude dedne bolezni ali če je to potrebno zaradi uspešnosti postopka oploditve z biomedicinsko pomočjo (OBMP). V Sloveniji se ti postopki izvajajo že od leta 2004 in to samo v primerih nevarnosti prenosa hude dedne bolezni na potomce. Tovrstnih preiskav ne izvajamo z namenom izboljšati uspešnost postopkov OBMP, saj tudi v svetovnem merilu še ni popolnoma jasno, v katerih primerih bi bilo to smiselno. Zaenkrat se zdi, da so primerne predvsem pri starejših ženskah, saj se lahko na ta način zmanjša pojavnost splava in skrajša čas do zanositve.(17) Postopke t.i. predimplantacijskega genetskega testiranja (PGT), ki jih izvajamo z namenom preprečitve rojstva otroka s hudo dedno boleznijo, lahko sicer podrobneje in natančneje razdelimo na PGT-M (angl. monogenic defects), pri katerem se zarodke testira za preprečevanje prenosa monogenskih bolezni in na PGT-SR (angl. structural rearrangements), pri katerem se zarodke pregleda za preprečevanje prenosa strukturnih kromosomskih nepravilnosti. Način preiskave, s katero se poskuša izboljšati uspešnost postopkov OBMP, se imenuje PGT-A (angl. aneuploidy); v tem priemeru se zarodke pregleda na število kromosomov oz. prisotnost aneuplodij.

Za genetski pregled zarodka je le-tega potrebno biopsirati. Biopsija se lahko izvede na stopnji manjceličnega zarodka na tretji dan razvoja ali pa na stopnji blastociste na 5/6 dan razvoja. Zarodek na tretji dan razvoja ima normalno od 6-8 celic, kar predstavlja omejitev pri količini DNK. Ob biopsiji v tej fazi razvoja, se iz zarodka odvzame 1 ali največ 2 celici (blastomeri). Raziskave kažejo, da odvzem 1-2 celic lahko hipotetično negativno vpliva na nadaljnji razvoj in implantacijski potencial zarodka. Slabost tega pristopa je tudi ta, da ni mogoče zanesljivo določiti mitotskih napak, ki vodijo v mozaicizem. Prednost postopka je v tem, da omogoča določitev mejotskih napak ali mutacij od obeh staršev.

Pristop, ki pa omogoča bolj zanesljive rezultate, je biopsija zarodka na stopnji blastociste. Takrat ima zarodek običajno okoli 100 celic, lahko pa se tudi že razloči del zarodka iz katerega se razvije plod (embrioblast) in del zarodka iz katerega se razvije placenta (trofoblast). Biopsija se izvede tako, da se s pomočjo laserja odreže manjši del trofoblasta (okoli 5-10 celic), zarodek pa se nato za čas izvedbe genestke preiskave zamrzne v tekočem dušiku. Prednost je tudi v tem, da se biopsirajo samo zarodki, ki imajo potencial za implantacijo. Slabost te metode je ta, da se v določenem deležu lahko pojavi mozaicizem, kar pa je manj problematično kot v primeru biopsije manjceličnih zarodkov. Kljub temu, da rezultati raziskav kažejo, da postopki PGT ne vplivajo slabo na zdravstveno stanje in razvoj otrok, se zaradi relativno invazivnega posega v zarodek ves čas pojavljajo pomisleki, da bi takšni postopki lahko negativno vplivali na zdravje otrok.(18)

Hkrati se nenehno išče manj invazivne in neinvazivne postopke za pridobitev genetskega materiala iz zarodka, saj bi se lahko z njimi izognili vsem pomislekom glede direktega posega vanj. Manj invaziven postopek od biopsije blastociste je biospija blastocela, s katero se posrka tekočina iz blastocela, kjer se nahaja tudi zarodkova DNK, blastocista pa med tem postopkom kolabira. Tak postopek ponekod uporabljajo celo v klinični praksi pred vitrifikacijo zarodkov, saj naj bi izboljšal preživetje blastociste, pa čeprav z njim na nek način vseeno posežemo v zarodek. Popolnoma neinvaziven postopek pa je genetska analiza gojišča, v katerem se goji zarodke. To je mogoče zato, ker zarodki med razvojem izločajo dele jedrne in mitohondrijske DNK, ki jo s sodobnimi molekularnimi metodami lahko zaznamo in nato določimo genetski status zarodka z vidika pravilnosti števila kromosomov. Nenormalno število kromosomov ob uspešni vgnezditvi zarodka običajno vodi v splav, s čimer se bolnica izpostavi nepotrebnemu stresu, ob tem se podaljša tudi čas do morebitne nove zanositve. Vse našteto je zelo neugodno za bolnice v poznem reproduktivnem obdobju ali z grozečo prezgodnjo ovarijsko odpovedjo.

Tako po podatkih Ginekološke klinike (19), kot tudi po rezultatih pravkar objavljene nizozemske raziskave (20), ZOJ pri mlajših bolnicah ne predstavlja večjega problema, saj je stopnja živorojenih otrok po postopkih zunajtelesne oploditve (ZTO) skoraj enaka kot pri dobro odzivnih pacientkah. Obravnava starejših pacientk z ZOJ pa predstavlja velik izziv.

2. Pregled in analiza dosedanjih raziskav in relevantne literature

Najbolj uspešno “zdravljenje“ za bolnice z ZOJ po nespešnih postopkih ZTO je uporaba darovanih jajčnih celic. Stopnja živorojenih otrok pri pacientkah z ZOJ se giblje med 1 do 10% na ciklus, Stopnja živorojenih otrok z darovanimi jajčnimi celicami pa med 50 in 70%.(3) Odločitev za darovane jajčne celice je za pare pogosto težko sprejemljiva, še zlasti takrat, ko je še možno pričakovati nosečnost z lastnimi jajčnimi celicami, čeprav z zelo majhno verjetnostjo. Zato so bolnice pripravljene tudi na večkratno ponovitev izvajanja postopkov. Zaradi navedenega, velik izziv za reproduktivne ginekologe predstavlja iskanje najboljšega oz. najučinkovitejšega protokola za spodbujanje jajčnikov v postopkih ZTO pri pacientkah z ZOJ. Številne do sedaj opravljene raziskave niso privedle do enotnih priporočil in odločitev.(3)

Pri primerjavi spodbujanja jajčnikov z gonadotropini v kombinaciji z agonisti ali antagonisti gonadoliberinov so potrdili boljše izide postopkov ZTO pri uporabi agonistov gonadoliberinov. Prekinitev postopkov je bilo manj (10 vs 20 %), stopnja implantacije je bila višja (25.3 vs 10.7 %), prav tako tudi stopnja živorojenih otrok (27.6 vs 13 %). Ti rezutati so veljali za mlade pacientke z ZOJ POSEIDON skupine 3, niso pa potrdili nobenih razlik pri uporabi agonistov ali antagonistov gonadoliberinov pri starejših pacientkah z ZOJ POSEIDON skupine 4.(21)

Tudi na Ginekološki kliniki v Ljubljani smo leta 2016 analizirali in objavili svoje lastne podatke o izidih postopkov ZTO pri pacientkah z ZOJ. Primerjali smo 142 postopkov ZTO (skupina agonistov gonadoliberinov), 53 postopkov ZTO (skupina antagonistov gonadoliberinov) in 36 postopkov ZTO v spontanih ciklusih. Povprečno število jajčnih celic (2.8±1.8) in zarodkov (1.6±1.2) na aspiracijo je bilo statistično pomembmo višje v skupini agonistov v primerjavi s skupino antagonistov gonadoliberinov in spontanih ciklusov. Delež nezrelih jajčnih celic, stopnja oploditve in delež zarodkov dobre kakovosti se med skupinami ni razlikoval. Prav tako stopnja zanositev se ni statistično pomembno razlikovala (18.9% po agonistih gonadoliberinov, 10.6% po antagonistih gonadoliberinov in 5.6% po spontanih ciklusih). Statistično pomembno višja pa je bila stopnja živorojenih otrok na aspiracijo foliklov v skupini agonistov gonadoliberinov v primerjavi z antagonisti gonadoliberinov (15.1% vs. 4.2%; p=0.024). Pri uporabi antagonistov gonadoliberinov predvidevamo, da je nižja stopnja živorojenih otrok posledica manj receptivnega endometrija zaradi njegove prezgodnje maturacije, ki nastopi pri podaljševanju spodbujanja jajčnikov z gonadotropini v folikularni fazi.(22)

Eden izmed poskusov pridobiti več jajčnih celic je spodbujanje jajčnikov z visokimi odmerki gonadotropinov, vendar dogovor med odločevalci do sedaj še ni bil dosežen in tudi v številnih študijah ni bil verodostojno podprt.(23) Vedno bolj pa je znano, da z višjimi odmerki sicer pridobimo kakšno jajčno celico več in znižamo stopnjo prekinjenih postopkov ZTO, ne zvišamo pa zbirne stopnje živorojenih otrok (24), ker na to pomembno vplivajo tudi starost pacientke, genetska kvaliteta jajčnih celic (25), lastnosti semena in vgnezditvena sposobnost endometrija.(26,27) Številne študije potrjujejo, da so jajčne celice, ki jih pridobimo z višanjem odmerkov gonadotropinov slabše kvalitete na račun večje jederne nezrelosti. To potrjuje dejstvo, da med antralnimi folikli obstaja določena hierarhija in njihova sposobnost odgovora na gonadotropine z najkvalitetnejšimi jajčnimi celicami.(23) Ta del razmišljanja vodi k odločitvi o kontinuiranem spodbujanju jajčnikov z namenom, da bi v celem menstruacijskemu ciklusi prišli do kohorte najperspektivnejših foliklov s kvalitetnimi jajčnimi celicami. Ubaldi s sod so v raziskavi, kjer so spodbujali jajčnike pri bolnicah z ZOJ tako v folikularni kot lutealni fazi v istem menstracijskem ciklusu, v obeh fazah pridobili enako število evploidnih blastocist po injiciranju MII jajčnih celic. Dodatno spodbujanje v lutealni fazi je tako pomembno prispevalo k večjem številu prenosov zarodkov in zelo visoko stopnjo rojstev - 66% na prenos evploidnih blastocyst.(28)

V času prvega vala epidemije COVID, smo bili zaradi praktičnih razlogov pri trinajstih starejših in onkoloških bolnicah primorani opraviti kontinuirano spodbujanje jajčnikov. Vse zarodke smo zamrznili, saj še nismo razpolagali s podatki, kako okužba s COVID vpliva na zarodke in predvsem na nosečnost. Analiza podatkov je pokazala, da smo v lutealni fazi v primerjavi s folikularno fazo pridobili celo nekoliko večje število jajčnih celic in zarodkov, vendar razlika zaradi majhnega števila vključenih bolnic ni dosegla statistične pomembnosti.

Naslednji sklop raziskav predstavljajo tiste, ki potrjujejo zvečano zbirno stopnjo živorojenih otrok po večkratnem ponavljanju postopkov ZTO pri bolnicah z ZOJ. Velika nizozemska raziskava je pri 551 bolnicahh z ZOJ in prenosom 1128 svežih in 329 odmrznjenih zarodkov v 18 mesečnem obdobju potrdila 56% skupno zbirno stopnjo živorojenih otrok. Ponovno se je izkazalo, da starost bolnic najbolj negativno vpliva na izid postopkov ZTO. Pri starejših pacientkah z nizko rezervo jajčnikov je bila zbirna stopnja živorojenih otrok 39%, pri mladih pa med 65 do 68%, kar je enak rezultat kot pri bolnicah z normalnim odzivom na spodbujanje jajčnikov.(29)

Tudi na Ginekološki kliniki v Ljubljani smo analizirali in objavili podatke 395 bolnic, pri katerih smo opravili 810 postopkov ZTO. Prvi postopek je potekal leta 2006, bolnice smo nato sledili vse do leta 2012. V analizi smo upoštevali vse ponovljene postopke ZTO in prenos vseh odmrznjenih zarodkov v tem obdobju. Zbirno stopnjo zanositev in rojstev smo izračunali glede na starost bolnic in ZOJ. Pri primerjavi žensk s ZOJ, starih manj kot 38 let in več kot 38 let, je bila zbirna stopnja živorojenih otrok statistično pomembno različna (50% vs 19%, p= 0.003).(19)

Glede na to, da bi lahko ločeno raziskovali reciptivnost endometrija in njegov prispevek k uspešni vgnezditvi, je pomembno vedeti, kakšen je status prenesenih zarodkov glede na evploidnost.

Namen PGT-A postopkov je ugotoviti, kateri zarodki imajo normalen genetski status z vidika števila kromosomov, saj te nepravilnosti (anevplodije) najpogosteje privedejo do spontanih splavov. Trenutno se uporablja invaziven pristop z biopsijo trofoektoderma zarodka. Ta postopek pa ima kljub dobremu namenu nekaj pomanjkljivosti in trenutno še nedokazan pozitiven učinek za neselekcionirano populacijo bolnic.(30) Glavna težava je zanesljivost rezultatov v smislu reprezentativnosti biopsiranega vzorca za cel zarodek. Največji izziv predstavlja mozaicizem, ki nastane zaradi napak med mitozo in pomeni prisotnost genetsko različnih celic v zarodku.(31) Te celice so lahko naključno razvrščene med normalne celice. Ni pa še popolnoma jasno, kolikšna je še normalna stopnja mozaicizma. V preteklosti se je dogajalo, da se je veliko število zarodkov, ki so bili diagnosticirani kot nenormalni-anevploidni zavrglo, vendar zadnji podatki kažejo, da temu verjetno ni bilo tako.(32) Druga pomanjkljivost določanja kromosomskega statusa zarodka iz celic trofoektoderma je tudi invazivnost postopka pridobitve teh celic, saj se jih pridobi z biopsijo, ki lahko škoduje zarodku.(33) Zato je neinvazivnost metode za pridobivanje genetskega materiala izrednega pomena. Tak način je analiza gojišča gojenih zarodkov. Dolgo časa tak pristop ni bil mogoč, saj se v gojišču nahaja relativno malo brezcelične DNK zarodka, metode za analizo pa imajo omejitve pri zaznavi DNK.(34,35) Z novimi pristopi teh težav ni več (36), raziskave pa potrjujejo, da so rezultati tovrstnega genetskega testiranja za prisotnost anevplodij lahko popolnoma primerljivi z rezultati klasične biopsije blastociste.(35) Predpostavljeno je bilo celo to, da je z analizo brezcelične DNK iz gojišča gojenega zarodka mogoče bolj reprezentativno določiti stopnjo mozaicizma za celoten zarodek, v primerjavi za klasično biopsijo.(37)

**Izbrana literatura:**

1. Oudendijk JF, Yarde F, Eijkemans MJ, Broekmans FJ, Broer SL.Hum Reprod Update. 2012 Jan-Feb;18(1):1-111.

2. Ubaldi F, Vaiarelli A, D’Anna R, Rienzi L. Management of poor respond- ers in IVF: is there anything new? Biomed Res Int 2014:352098

3. Blumenfeld Z. [What Is the Best Regimen for Ovarian Stimulation of Poor Responders in ART/IVF?](https://pubmed.ncbi.nlm.nih.gov/32362870/) Front Endocrinol 2020 Apr 17;11:192

4. Polyzos NP, Devroey P. A systematic review of randomized trials for the treat- ment of poor ovarian responders: is there any light at the end of the tunnel? Fertil Steril 2011;96:1058–61.

5. Poseidon Group (Patient-Oriented Strategies Encompassing IndividualizeD Oocyte Number), Alviggi C, Andersen CY, Buehler K, Conforti A, De Placido G, et al. A new more detailed stratification of low responders to ovarian stimu- lation: from a poor ovarian response to a low prognosis concept. Fertil Steril 2016;105:1452–3.

6. Ata B, Kaplan B, Danzer H, Glassner M, Opsahl M, Tan SL, et al. Array CGH analysis shows that aneuploidy is not related to the number of embryos gener- ated. Reprod Biomed Online 2012; 24:614–20.

7. Patrizio P, Vaiarelli A, Levi Setti PE, Tobler KJ, Shoham G, Leong M, et al. How to define, diagnose and treat poor responders? Responses from a worldwide survey of IVF clinics. Reprod Biomed Online 2015;30:581–92.

8. Iliodromiti S, Anderson RA, Nelson SM. Technical and performance char- acteristics of anti-Mullerian hormone and antral follicle count as biomarkers of ovarian response. Hum Reprod Update 2015;21:698–710.

9. Busnelli A, Papaleo E, Del Prato D, La Vecchia I, Iachini E, Paffoni A, et al. A retrospective evaluation of prognosis and cost-effectiveness of IVF in poor responders according to the Bologna criteria. Hum Reprod 2015;30:315–22.

10. Baerwald AR, Adams GP, Pierson RA. Ovarian antral folliculogenesis during the human menstrual cycle: a review. Hum Reprod Update 2012;18:73–91.

11. Kuang Y, Chen Q, Hong Q, Lyu Q, Ai A, Fu Y, et al. Double stimulations during the follicular and luteal phases of poor responders in IVF/ICSI programmes (Shanghai protocol). Reprod Biomed Online 2014;29:684–91.

12. Shapiro BS, Daneshmand ST, Garner FC, Aguirre M, Hudson C. Clinical rationale for cryopreservation of entire embryo cohorts in lieu of fresh transfer. Fertil Steril 2014;102:3–9.

13. Wang A, Santistevan A, Hunter Cohn K, Copperman A, Nulsen J, Miller BT, Widra E, Westphal LM, Yurttas Beim P. Freeze-only versus fresh embryo transfer in a multicenter matched cohort study: contribution of progesterone and maternal age to success rates. Fertil Steril 2017 Aug;108(2):254-261.

14. Shapiro BS, Daneshmand ST, Garner FC, Aguirre M, Hudson C, Thomas S. Evidence of impaired endometrial receptivity after ovarian stimulation for in vitro fertilization: a prospective randomized trial comparing fresh and frozen-thawed embryo transfer in normal responders. Fertil Steril 2011; 96: 344–348.

15. Horcajadas JA, Riesewijk A, Polman J, van Os R, Pellicer A, Mosselman S, et al. Effect of controlled ovarian hyperstimulation in IVF on endometrial gene expression profiles. Mol Hum Reprod 2005;11:195–205.

16. Huang R, Fang C, Xu S, Yi Y, Liang X. Premature progesterone rise negatively correlated with live birth rate in IVF cycles with GnRH agonist: an analysis of 2,566 cycles. Fertil Steril 2012;98:664–70.

17. Kang HJ, Melnick AP, Stewart JD, Xu K, Rosenwaks Z. Preimplantation genetic screening: who benefits?. Fertil Steril. 2016;106(3):597-602.

18. Greco, E., Greco, A., & Minasi, M. G. (2019). Reassuring data concerning follow-up data of children born after preimplantation genetic diagnosis. Fertility and sterility, 111(6), 1111–1112.

19. Vrtačnik U, Vrtačnik Bokal E, Devjak R.Cumulative Delivery Rate after Providing Full Reimbursement In Vitro Fertilization Programme: A 6-Years Survey. Biomed Res Int 2014

20. Leijdekkers JA, Eijkemans MJC, van Tilborg TC, Oudshoorn SC, van Golde RJT, Hoek A, Lambalk CB, de Bruin JP, Fleischer K, Mochtar MH, Kuchenbecker WKH, Laven JSE, Mol BWJ, Torrance HL, Broekmans FJM; OPTIMIST study group. [Cumulative live birth rates in low-prognosis women.](https://pubmed.ncbi.nlm.nih.gov/31125412/) Hum Reprod. 2019 Jun 4;34(6):1030-1041.

21. Huang MC, Tzeng SL, Lee CI, Chen HH , Huang CC, Lee TH, Lee MS. GnRH agonist long protocol versus GnRH antagonist protocol for various aged patients with diminished ovarian reserve: A retrospective study PLoS ONE 2018 Nov 7;13(11):e0207081

22. Stimpfel M, Vrtačnik-Bokal E, Pozlep B, Kmecl J, Virant-Klun I. Gonadotrophin-releasing hormone agonist protocol of controlled ovarian hyperstimulation as an efficient treatment in Bologna-defined poor ovarian responders. Syst Biol Reprod Med. 2016 Aug;62(4):290-6.

23. Jori A Leijdekkers, Helen L Torrance, Nienke E Schouten, Theodora C van Tilborg, Simone C Oudshoorn, Ben Willem J Mol, Marinus J C Eijkemans, [Frank J M Broekmans](https://pubmed.ncbi.nlm.nih.gov/?term=Broekmans+FJM&cauthor_id=31838515). Individualized ovarian stimulation in IVF/ICSI treatment: it is time to stop using high FSH doses in predicted low responders. Hum Reprod 2020 Sep 1;35(9):1954-1963.

24. Van Tilborg TC, Torrance HL, Oudshoorn SC, Eijkemans MJC, Koks CAM, Verhoeve HR, Nap AW, Scheffer GJ, Manger AP, Schoot BC, Sluijmer AV, Verhoeff A, Groen H, Laven JSE, Mol BWJ, Broekmans FJM. OPTIMIST study group. [Individualized versus standard FSH dosing in women starting IVF/ICSI: an RCT. Part 1: The predicted poor responder.](https://pubmed.ncbi.nlm.nih.gov/29121326/) Hum Reprod. 2017 Dec 1;32(12):2496-2505.

25. Broekmans FJ, Knauff EA, te Velde ER, Macklon NS, Fauser BC. [Female reproductive ageing: current knowledge and future trends.](https://pubmed.ncbi.nlm.nih.gov/17275321/) Trends Endocrinol Metab. 2007 Mar;18(2):58-65.

26. Simon L, Murphy K, Shamsi MB, Liu L, Emery B, Aston KI, Hotaling J, Carrell DT. [Paternal influence of sperm DNA integrity on early embryonic development.](https://pubmed.ncbi.nlm.nih.gov/25205757/) Hum Reprod. 2014 Nov;29(11):2402-12.

27. Liu KE, Hartman M, Hartman A, Luo ZC, Mahutte N. The impact of a thin endometrial lining on fresh and frozen-thaw IVF outcomes: an analysis of over 40 000 embryo transfer. Hum Reprod 2018:33; 1883-1888.

28. Ubaldi FM, Capalbo A, Vaiarelli A, Cimadomo D, Colamaria S, Alviggi C, Trabucco E, Venturella R, Vajta G, Rienzi L. [Follicular versus luteal phase ovarian stimulation during the same menstrual cycle (DuoStim) in a reduced ovarian reserve population results in a similar euploid blastocyst formation rate: new insight in ovarian reserve exploitation.](https://pubmed.ncbi.nlm.nih.gov/27020168/) Fertil Steril. 2016 Jun;105(6):1488-1495.

29. Leijdekkers JA, Eijkemans MJC, van Tilborg TC, Oudshoorn SC, van Golde RJT, Hoek A, Lambalk CB, de Bruin JP, Fleischer K, Mochtar MH, Kuchenbecker WKH, Laven JSE, Mol BWJ, Torrance HL, Broekmans FJM; OPTIMIST study group. [Cumulative live birth rates in low-prognosis women.](https://pubmed.ncbi.nlm.nih.gov/31125412/) Hum Reprod. 2019 Jun 4;34(6):1030-1041

30. Munné S, Kaplan B, Frattarelli JL, et al. Preimplantation genetic testing for aneuploidy versus morphology as selection criteria for single frozen-thawed embryo transfer in good-prognosis patients: a multicenter randomized clinical trial. Fertil Steril. 2019;112(6):1071-1079.e7. doi:10.1016/j.fertnstert.2019.07.1346

31. Popovic M, Dhaenens L, Boel A, Menten B, Heindryckx B. Chromosomal mosaicism in human blastocysts: the ultimate diagnostic dilemma [published correction appears in Hum Reprod Update. 2020 Apr 15;26(3):450-451]. Hum Reprod Update. 2020;26(3):313-334. doi:10.1093/humupd/dmz050

32. Gleicher N, Kushnir VA, Barad DH. How PGS/PGT-A laboratories succeeded in losing all credibility. Reprod Biomed Online. 2018;37(2):242-245. doi:10.1016/j.rbmo.2018.06.019

33. Leaver M, Wells D. Non-invasive preimplantation genetic testing (niPGT): the next revolution in reproductive genetics? Hum Reprod Update. 2020;26(1):16-42. doi:10.1093/humupd/dmz033

34. Shamonki MI, Jin H, Haimowitz Z, Liu L. Proof of concept: preimplantation genetic screening without embryo biopsy through analysis of cell-free DNA in spent embryo culture media. Fertil Steril. 2016;106(6):1312-1318. doi:10.1016/j.fertnstert.2016.07.1112

35. Kuznyetsov V, Madjunkova S, Abramov R, et al. Minimally Invasive Cell-Free Human Embryo Aneuploidy Testing (miPGT-A) Utilizing Combined Spent Embryo Culture Medium and Blastocoel Fluid -Towards Development of a Clinical Assay. Sci Rep. 2020;10(1):7244. Published 2020 Apr 29. doi:10.1038/s41598-020-64335-3

36. Babariya D. Non-invasive preimplantation genetic testing for aneuploidy (NI PGT-A). Reprod Biomed Online. 2019;38(Suppl 1):e10-e11. Doi.org/10.1016/j.rbmo.2019.03.020

37. Huang L, Bogale B, Tang Y, Lu S, Xie XS, Racowsky C. Noninvasive preimplantation genetic testing for aneuploidy in spent medium may be more reliable than trophectoderm biopsy. Proc Natl Acad Sci U S A. 2019;116(28):14105-14112. doi:10.1073/pnas.1907472116

38. Chandramouli K, Qian PY. Proteomics: challenges, techniques and possibilities to overcome biological sample complexity. Hum Genomics Proteomics. 2009;2009:239204. Published 2009 Dec 8. doi:10.4061/2009/239204

39. Janša V, Klančič T, Vrtačnik Bokal E, Ban Frangež H, Lanišnik Rižner T. Proteomic analyses of peritoneal fluid cartilage oligomeric matrix protein and transforming growth factor-beta-induced protein ig-h3 as new candidate biomarkers for endometriosis. The article is under revision.

40. Abdulkhalikova D, Jančar N, Jensterle M, Šuštaršič A, Bokal Vrtačnik E. The influence of lifestyle modifications on anthropometric, metabolic and reproductive outcomes and endometrial proteome of women with polycystic ovarian syndrome and obesity. The article is under revision.

3. Namen raziskave

- Predvidevamo, da bomo s kontinuiranim spodbujanjem jajčnikov (tako v folikularni kot lutealni fazi) pridobili večje število jajčnih celic v enem postopku kot v prejšnjih dveh postopkih skupaj, kjer smo izvajali spodbujanje jajčnikov samo v folikularni fazi. Hkrati domnevamo, da bomo z večjo kohorto vzpodbujenih foliklov imeli tudi večjo verjetnost pridobitve kvalitetnejših jajčnih celic, kar bo vodilo v večje število zarodkov dobre kvalitete. Zamrzovanje vseh zarodkov nam bo omogočilo podaljšano spodbujanje jajčnikov z gonadotropini, ker se ne bomo izogibali porasta progesterona in posledične prezgodnje maturacije endometrija, ki negativno vpliva na vgnezditev zarodka.
- Ženske, pri katerih je potrebno večkratno ponavljanje postopkov ZTO, zaradi psihičnih obremenitev večkrat odstopijo od nadaljevanja zdravljenja, zato bi kontinuirano spodbujanje predstavljajo manjšo in krajšo obremenitev bolnic. Običajno med zaključenim in novim postopkom preteče 3 mesece, pri kontinuiranem spodbujanju pa bi bil postopek opravljen v 1 mesecu, kar je še posebno pomembno za starejše bolnice.
- Naslednji cilj je ločevanje med evploidnimi in anevploidnimi zarodki na osnovi neinvazivne DNK analize iz gojišča. Na ta način bi se izognili morebitnim poškodbam zarodka po opravljeni biopsiji ektoderma, ki je potrebna za izvedbo PGT.
- Predvidevamo, da bomo uspeli določiti profil proteinov, značilen za receptivnost v endometriju s primerjavo proteomov v spontanih in spodbujenih ciklusih ter s primerjavo proteomov tistih žensk, ki so zanosile in tistih, ki niso. Tako bi lahko potrdili klinična predvidevanja, da je zamrzovanje in nato prenos zarodkov v spontanem ciklusu primernejši način za pacientke z ZOJ.

4. Osebe, ki bodo povabljene v raziskavo

V predlagano raziskavo bomo prospektivno vključili vse zaporedne ženske z ZOJ na spodbujanje z gonadotropini pred dopolnjenim 43. letom starosti, ki bodo opravljale postopek ZTO na Kliničnem oddelku za reprodukcijo Ginekološke klinike v Ljubljani, pri katerih po dveh postopkih ZTO ni prišlo do zanositve in rojstva. Predvidoma bomo vključili 100 pacientk v dvoletnem obdobju. Izključile bomo vse ženske, ki so zdravljene s postopki ZTO zaradi hudega moškega vzroka neplodnosti.

Ob vključitvi v raziskavo bomo preiskovane pare ustrezno informirali o načrtu in cilju raziskave. Vse ženske bodo podpisali pisno privolitev za sodelovanje v raziskavi (v prilogi). Izjavljamo, da bo pristanek za vključitev v raziskavo svobodna izbira preiskovanke in da vabljenje k sodelovanju ne bo spremljal pritisk in neprimerno napeljevanje. Bolnice, ki v raziskavi ne bodo želele sodelovati, bodo deležne enake obravnave kot sicer.

5. Metode

Kljub temu, da do sedaj še ni bila sprejeta enotna definicija za ZOJ, je največkrat uporabljena Bolonjska klasifikacija. Ta definira ZOJ s starostjo pacientke, slabim odzivom na spodbujanje jajčnikov v prejšnjih postopkih, z zmanjšanim številom antralnih foliklov (AFC) ter znižano serumsko koncentracijo anti-Mullerjevega hormona (AMH).

V predlagani raziskavi bomo pacientke razvrstili po zadnji POSEIDON klasifikaciji (Patient Oriented Strategies Encompassing Individualized Oocyte Number) (5), ki upošteva starost bolnice, pričakovano stopnjo anevploidij pri zarodkih, označevalce ovarijske rezerve (AMH, AFC) in odgovor na spodujanje jajčnikov v prejšnjem postopku ZTO.

Bolnice bomo razdelili v 4 skupine:

POSEIDON skupina 1: Bolnice, mlajše od 35 let z normalnimi označevalci ovarijske rezerve (AMH>1.2ng/mL, AFC >5) in nepričakovanim ZOJ (podskupini: 1a: <4 pridobljene jajčne celice (JC); 1b: 4-9 pridobljenih JC).

POSEIDON skupina 2: Bolnice, starejše od 35 let z normalnimi označevalci ovarijske rezerve (AMH>1.2ng/mL, AFC>5) in nepričakovanim ZOJ (podskupini: 1a: <4 pridobljene JC; 1b: 4-9 pridobljenih JC).

POSEIDON skupina 3: Bolnice, mlajše od 35 let z ZOJ (AMH<1.2ng/mL, AFC<5).

POSEIDON skupina 4: Bolnice, starejše od 35 let z ZOJ (AMH<1.2ng/mL, AFC<5).

Pri vseh bolnicah bomo uvedli enoten protokol s spodujanjem jajčnikov v folikularni in lutealni fazi menstruacijskega ciklusa. Enaindvajseti dan ciklusa bomo uvedli 4 mg estradiol valerata za sinhronizacijo in koordinacijo rasti foliklov.(10) Na dan 2 naslednjega menstruacijskega ciklusa bomo prekinili z estradiol valeratom in pričeli spodbujanje z rFSH 300 IU/dan. Z antagonisti gonadoliberinov bomo pričeli 7. dan ciklusa, tako v folikularni kot v lutealni fazi. Za zorenje jajčnih celic bomo uporabili GnRH agonist (Gonapeptyl 0.1 mg sc) in sicer takrat, ko folikli dosežejo velikost 17-18 mm v premeru. Aspiracijo foliklov bomo opravili 36 ur po vnosu agonista. Pet dni po prvi aspiraciji foliklov, ko bo nastopila popolna luteoliza, bomo pričeli spodbujanje na enak način kot v folikularni fazi. Oploditev, kultiviranje do blastociste in vitrifikacijo bomo izvedli po ustaljenih laboratorijskih metodah. Blastociste bomo po odmrznenju zarodkov prenašali v naslednjem spontanem ali spodbujenem ciklusu. Gojišča, v katerih bomo gojili zarodke, bomo shranili na -80 st, za kasnejšo neinvazivno PGT-A. Novo metodo bomo razvili in najprej potrdili na kliničnem PGT programu. Na ta način bomo lahko ločeno ugotovili kakšen vpliv ima endometrij oz. kvaliteta zarodkov na stopnjo zanositev po postopkih ZTO bolnic z ZOJ.

Neinvazivno metodo določitve kromosomskega statusa zarodka po izolaciji DNK iz gojišča, v katerem bo zarodek gojen, bomo najprej validirali pri zarodkih v rednem kliničnem PGT programu. Šele nato bomo z neinvazivnim pristopom analizirali kromosomski status zarodkov pri naši ciljni populaciji bolnic z ZOJ. Natančneje to pomeni, da bomo zarodke v PGT-SR programu gojili na enak način, kot jih tudi sicer gojimo v klinični praksi (3. dan predimplantacijskega razvoja individualno gojenje v kapljicah z volumnom 40 mikrolitrov). Na ta dan z laserjem naredimo odprtino v zoni pelucidi zarodka - tako se blastocista lahko lažje levi in zato tudi lažje biopsiramo fragment trofoblasta za genetsko analizo. Za primerjavo z invazivnim postopkom bomo tik pred biopsijo odvzeli 10 mikrolitrov gojišča, v katerem se zarodek goji in ga nato genetsko testirali. Po potrjeni zanesljivosti rezultatov, bomo uporabili enako metodo pri naši ciljni populaciji bolnic z ZOJ. Zarodke teh bolnic bomo gojili popolnoma enako kot sicer. Edina razlika bo le v tem, da bomo te zarodke od 3. dneva naprej gojili individualno v kapljicah gojišča. Na 3. dan bomo zarodke tudi temeljito sprali s svežim gojiščem, z namenom da bi z njihove površine odstranili morebitne ostanke DNK granuloza celic ali spermijev, ki bi lahko v analizi dali popačene podatke.

Predvidevamo, da pri bolnicah z ZOJ na nizko stopnjo zanositev vpliva tako zmanjšano število pridobljenih MII celic z večjim številom anevploidnih zarodkov, kot tudi spremenjena receptivnost endometrija, zato bomo za oceno receptivnosti endometrija določali profil izražanja proteinov v času predvidenega vgnezditvenega okna. Pri ženskah s kontinuiranim spodbujanjem bomo vzorec endometrija in izpirek maternične votline odvzeli pred vstopom v postopek ZTO, 21. do 23. dan menstruacijskega ciklusa in enak postopek ponovili 5 dni po prvi aspiraciji foliklov oz. neposredno pred pričetkom spodbujanja v lutealni fazi. To je čas, primeren za prenos zarodkov v svežih postopkih ZTO. Pri izvajanju kontinuiranega spodbujanja bomo pridobili informacijo, kako se profil proteinov v endometriju vgnezditvenega okna spontanega menstruacijskega ciklusa razlikuje od profila proteinov v endometriju po spodbujanju z visokimi odmerki gonadotropinov, kar lahko posledično vpliva na vgnezditev in razvoj zarodka. Profil proteinov bomo določali s proteinskimi mrežami, ki omogočajo visoko zmogljive tarčne študije večjega nabora proteinov v različnih bioloških vzorcih.(38). Ta nova tehnologija je nadvse primerna za iskanje novih biokemijskih označevalcev. V naši prejšnji raziskavi smo uporabili proteinsko mrežo (Sciomics GmbH, Heidelberg, Germany), ki lahko določi 1438 različnih proteinov s 1925 protitelesi in se je izkazala za primerno za analizo profila proteinov v endometriju, zato bomo tudi tokrat poslali vzorce v omenjeni laboratorij.(39,40)

Profila endometrija zaradi visoke cene ne bomo mogli opraviti pri vseh preiskovankah. Predvidevamo, da bomo lahko določili vgnezditveno okno na osnovi sklepanja iz analize profila proteinov pri 30 bolnicah. Iz POSEIDON skupine 1 bomo opravili odvzem petim bolnicam iz podskupine 1a in petim bolnicam iz podskupine 1b. Na enak način bomo opravili odvzem tudi bolnicam iz skupine 2. Iz skupine 3 in 4 pa bomo opravili odvzem petim ženskam iz vsake skupine. Pri vsaki bolnici bomo z mikromrežami analizirali proteine 2 krat, in sicer v spontanem in spodbujenem ciklusu.

Za oceno profila izražanja beljakovin v spontanih menstruacijskih ciklusih pri vseh preiskovankah bomo opravili prva biopsija endometrija v obdobju implantacijskega okna (20.–24. dan). Natančen dan ovulacije bo določen z ovulacijskimi testi. Vzorci bodozbrani s plastično pipelo (Rampipella Ri.Mos.S.R.L. Mirandola, Italija), hitro zamrznjeni in shranjeni pri -80 °C do končne analize.

Druga biopsija endometrija bo opravljena pet dni po prvi aspiraciji jajčnih celic (LH+7).

Ob biopsiji endometrija bomo delček sluznice poslali tudi na histopatološko preiskavo za izključevanje morebitnega kroničnega vnetja v maternični votlini, kar bi lahko negativno vplivalo na izid postopkov.

Z neinvazivno metodo določitve kromosomskega statusa zarodka po izolaciji DNK iz gojišča (PGT-A), v katerem bo zarodek gojen, bomo v sodelovanju z genetiki poskusili vzpostaviti novo metodo določevanja evploidnih blastocist. Metoda je tudi v svetu še v razvijanju in preverjanju. Prednost predvidene metode v primerjavi z biopsijo trofektoderma je ta, da se izognemo možni poškodbi zarodka. Selekcija blastocist bi temeljila na analizi pomnožene DNK v gojišču.

Primarni izidi raziskave bodo:

- Število jajčnih celic
- Število MII jajčnih celic
- Stopnja fertilizacije
- Število zarodkov
- Število zarodkov visoke kakovosti
- Stopnja zanositve

Vse navedene izide (1-6) bomo ocenjevali s primerjavo rezultatov kontinuiranega spodbujanja s prejšnjima postopkoma ZTO skupaj.

- Razlika v profilu proteinov v endometriju med spontanim in spodbujenim ciklusom
- Razlika v profilu proteinov v endometriju med ženskami, ki bodo zanosile in tistimi, ki ne bodo zanosile.

Sekundarni izidi raziskave:

- Delež anevplodnih zarodkov pri pacientkah z ZOJ, mlajših od 35 let
- Delež anevploidnih zarodkov pri pacientkah z ZOJ, starejših od 35 let
- Povprečna poraba gonadotropinov na eno jajčno celico v prejšnjih dveh opravljenih postopkih ZTO v primerjavi s kontinuiranim postopkom.

Statistična obdelava podatkov:

Uporabljali bomo metode deskriptivne statistike. Od statističnih testov bomo uporabili metode logistične regresije, test Mann Whitney in v kolikor bo potrebno Fisherjev eksaktni test. Statistično pomembnost korelacije med koncentracijami označevalcev bomo ugotavljali s Spearman Rank korelacijsko analizo. O statistični značilnosti rezultatov bomo govorili pri p <0.05. Za analizo bo uporabljena programska oprema SPSS verzija 19.0, po potrebi tudi druga programska oprema.

6. Povzetek načrta raziskave

Pri zdravljenju nepolodnosti s postopki zunajtelesne oploditve (ZTO) včasih naletimo na problem, povezan z zmanjšano odzivnostjo jajčnikov na stimulacijo. Prognoza bolnic z zmanjšano odzivnostjo jajčnikov je v veliki meri odvisna od starosti bolnice in števila pridobljenih jajčnih celic. Podatki iz literature kažejo, da zmanjšan odziv jajčnikov nastopi pri 9-24% neplodnih žensk. Prevalenca se še zlasti povečuje zaradi odmikanja nosečnosti v pozna trideseta leta ali celo štirideseta leta. Pri približno polovici teh pacientk vzrok ni znan. V želji po zvišanju stopnje živorojenih otrok se uporabljajo številni protokoli spodbujanja jajčnikov.

Dejstvo je, da število pridobljenih jajčnih celic in starost ženske najbolj vplivata na uspešnost zanositve. Torej je napovedovanje odziva jajčnikov z individualnim načrtovanjem spodbujanja zelo pomembno, še zlasti pri pacientkah z zmanjšanim odzivom jajčnikov. Trenutno nimamo dovolj dokazov, ki bi potrjevali prednost katerega od različnih znanih protokolov spodbujanja pri ženskah z zmanjšanim odzivom jajčnikov. Znano je le to, da je stopnja živorojenih otrok pri njih nižja v primerjavi z neplodnimi ženskami z normalnim ali zvišanim odzivom jajčnikov na stimulacijo.

V zadnjem času so znanstveniki prišli do spoznanja, da se v enem menstrualnem ciklusu (tudi v drugi polovici) rekrutira več foliklov in v več valovih, kot je bilo prej mišljeno. To odkritje je vodilo do razmišljanja, da bi z dvojnim spodbujanjem jajčnikov – tako v prvi, kot v drugi polovici menstrualnega ciklusa – lahko pridobili večje število jajčnih celic in zarodkov v enem menstruacijskem ciklusu. Zarodke bi nato zamrznili in jih prenesli v naslednjih ciklusih, saj vemo, da je stopnja zanositve s svežimi ali odmrznjenimi zarodki primerljiva. Prvo izkušnjo z dvojnim spodbujanjem je opisal Kuang s sodelavci, ki je poročal o enakem številu in razvojnem potencialu pridobljenih jajčnih celic v prvi in drugi fazi ciklusa. V času prvega vala epidemije COVID-19 smo tako ravnali tudi na Ginekološki kliniki v Ljubljani pri starejših ženskah in pridobili v drugi fazi celo nekaj več celic v primerjavi s prvo.

Glede na to, da je za uspešno oploditev potrebno medsebojno sinhrono delovanje sprejemljive maternične sluznice in zarodkov visoke kakovosti z normalnim številom kromosomov, se trenutne raziskave vedno bolj usmerjajo tudi v analizo profila proteinov v maternični sluznici z namenom prepoznavanja in določitve optimalnega časa za vgnezditev zarodka. Predvideva se, da izpostavljenost spodbujanju jajčnikov z zdravili in posledični spremenjeni ravni hormonov poslabša sprejemljivost maternične sluznice za zarodek (receptivnost), kar zmanjša stopnjo zanositve zlasti pri zarodkih slabše kakovosti. Vedno več je dokazov, da je sluznica maternice v ciklusih prenosa odmrznjenih zarodkov (torej brez stimulacije z zdravili) bolj receptivna v primerjavi s sluznico po spodbujanju jajčnikov. Hormonska regulacija ima pomembno vlogo pri receptivnosti sluznice endometrija, visoka koncentracija estrogena in progesterona pa lahko vpliva na izražanje genov in proteinov, vključenih v proces vgnezditve zarodkov. Asinhronija med zarodkom in nereceptivno sluznico maternice je lahko posledica spodbujanja jajčnikov, ki povzroči prezgodnji porast progesterona in posledično prezgodnje dozorevanje sluznice, kar zmoti obdobje vgnezditvenega okna, ki je optimalni čas za implantacijo zarodka.

Zakon o zdravljenju neplodnosti in postopkih oploditve z biomedicinsko pomočjo v 31. členu narekuje, da se genska preiskava zgodnjega zarodka sme opraviti samo v primeru nevarnosti hude dedne bolezni ali če je to potrebno zaradi uspešnosti postopka oploditve z biomedicinsko pomočjo (OBMP). V Sloveniji se ti postopki izvajajo že od leta 2004 in to samo v primerih nevarnosti prenosa hude dedne bolezni na potomce. Tovrstnih preiskav ne izvajamo z namenom izboljšati uspešnost postopkov OBMP, saj tudi v svetovnem merilu še ni popolnoma jasno, v katerih primerih bi bilo to smiselno. Zaenkrat se zdi, da so primerne predvsem pri starejših ženskah, saj se lahko na ta način zmanjša pojavnost splava in skrajša čas do zanositve. Postopke t.i. predimplantacijskega genetskega testiranja (PGT), ki jih izvajamo z namenom preprečitve rojstva otroka s hudo dedno boleznijo, lahko sicer podrobneje in natančneje razdelimo na PGT-M (angl. monogenic defects), pri katerem se zarodke testira za preprečevanje prenosa monogenskih bolezni in na PGT-SR (angl. structural rearrangements), pri katerem se zarodke pregleda za preprečevanje prenosa strukturnih kromosomskih nepravilnosti. Način preiskave, s katero se poskuša izboljšati uspešnost postopkov OBMP, se imenuje PGT-A (angl. aneuploidy); v tem priemeru se zarodke pregleda na število kromosomov oz. prisotnost aneuplodij.

Za genetski pregled zarodka je le-tega potrebno biopsirati. Biopsija se lahko izvede na stopnji manjceličnega zarodka na tretji dan razvoja ali pa na stopnji blastociste na 5/6 dan razvoja. Zarodek na tretji dan razvoja ima normalno od 6-8 celic, kar predstavlja omejitev pri količini DNK. Ob biopsiji v tej fazi razvoja, se iz zarodka odvzame 1 ali največ 2 celici (blastomeri). Raziskave kažejo, da odvzem 1-2 celic lahko hipotetično negativno vpliva na nadaljnji razvoj in implantacijski potencial zarodka. Slabost tega pristopa je tudi ta, da ni mogoče zanesljivo določiti mitotskih napak, ki vodijo v mozaicizem. Prednost postopka je v tem, da omogoča določitev mejotskih napak ali mutacij od obeh staršev.

Pristop, ki pa omogoča bolj zanesljive rezultate, je biopsija zarodka na stopnji blastociste. Takrat ima zarodek običajno okoli 100 celic, lahko pa se tudi že razloči del zarodka iz katerega se razvije plod (embrioblast) in del zarodka iz katerega se razvije placenta (trofoblast). Biopsija se izvede tako, da se s pomočjo laserja odreže manjši del trofoblasta (okoli 5-10 celic), zarodek pa se nato za čas izvedbe genestke preiskave zamrzne v tekočem dušiku. Prednost je tudi v tem, da se biopsirajo samo zarodki, ki imajo potencial za implantacijo. Slabost te metode je ta, da se v določenem deležu lahko pojavi mozaicizem, kar pa je manj problematično kot v primeru biopsije manjceličnih zarodkov. Kljub temu, da rezultati raziskav kažejo, da postopki PGT ne vplivajo slabo na zdravstveno stanje in razvoj otrok, se zaradi relativno invazivnega posega v zarodek ves čas pojavljajo pomisleki, da bi takšni postopki lahko negativno vplivali na zdravje otrok.

Hkrati se nenehno išče manj invazivne in neinvazivne postopke za pridobitev genetskega materiala iz zarodka, saj bi se lahko z njimi izognili vsem pomislekom glede direktega posega vanj. Manj invaziven postopek od biopsije blastociste je biospija blastocela, s katero se posrka tekočina iz votline blastociste (blastocela), kjer se nahaja tudi zarodkova DNK, blastocista pa med tem postopkom kolabira. Tak postopek ponekod uporabljajo celo v klinični praksi pred zamrzovanjem zarodkov, saj naj bi izboljšal preživetje blastociste, pa čeprav z njim na nek način vseeno posežemo v zarodek. Popolnoma neinvaziven postopek pa je genetska analiza gojišča, v katerem se goji zarodke. To je mogoče zato, ker zarodki med razvojem izločajo dele jedrne in mitohondrijske DNK, ki jo s sodobnimi molekularnimi metodami lahko zaznamo in nato določimo genetski status zarodka z vidika pravilnosti števila kromosomov. Nenormalno število kromosomov ob uspešni vgnezditvi zarodka običajno vodi v splav, s čimer se bolnica izpostavi nepotrebnemu stresu, ob tem se podaljša tudi čas do morebitne nove zanositve. Vse našteto je zelo neugodno za bolnice v poznem reproduktivnem obdobju ali z grozečo prezgodnjo ovarijsko odpovedjo.

Na Kliničnem oddleku za reprodukcijo Ginekološke klinike v Ljubljani načrtujemo klinično raziskavo, s katero želimo optimizirati postopek OBMP za bolnice z znanim zmanjšanim odzivom jajčnikov. Glede na podatke iz literature, bomo v tej skupini bolnic opravili postopek z dvojno stimulacijo jajčnikov – tako v folikularni, kot v lutelani fazi ciklusa. Hkrati bomo poskusili oceniti receptivnost maternične sluznice v naravnih menstruacijskih ciklusih in po stimulaciji jajčnikov in tako določiti beljakovine, ki imajo ključno vlogo pri določitvi optimalnega časa za vgnezditev zarodkov. Ob tem bomo poskusili vpeljati neinvazivno metodo za določitev kromoskomskega statusa zarodka z analizo gojišča, v katerem so bili zarodki gojeni.

V predlagano raziskavo bomo prospektivno vključili vse zaporedne ženske z zmanjšanim odzivom jajčnikov na spodbujanje z zdravili, ki bodo opravljale postopek zunajtelesne oploditve na Kliničnem oddelku za reprodukcijo Ginekološke klinike v Ljubljani, pri katerih po dveh postopkih ni prišlo do zanositve in rojstva. Predvidoma bomo vključili 100 pacientk v dvoletnem obdobju.

V predlagani raziskavi bomo bolnice razvrstili po zadnji POSEIDON klasifikaciji (Patient Oriented Strategies Encompassing Individualized Oocyte Number), ki upošteva starost bolnice, pričakovano stopnjo odklona v številu kromosomov pri zarodkih, označevalce rezerve jajčnikov (število antralnih foliklov (AFC), koncentracija anti-Mullerjevega hormona (AMH)) in odgovor na spodujanje jajčnikov v prejšnjem postopku zunajtelesne oplodtve.

Bolnice bomo razdelili v 4 skupine:

POSEIDON skupina 1: Bolnice, mlajše od 35 let z normalnimi označevalci rezerve jajčnikov (AMH>1.2ng/mL, AFC >5) in nepričakovanim zmanjšanim odzivom jajčnikov (podskupini: 1a: <4 pridobljene jajčne celice (JC); 1b: 4-9 pridobljenih JC).

POSEIDON skupina 2: Bolnice, starejše od 35 let z normalnimi označevalci rezerve jajčnikov (AMH>1.2ng/mL, AFC>5) in nepričakovanim zmanjšanim odzivom jajčnikov (podskupini: 1a: <4 pridobljene JC; 1b: 4-9 pridobljenih JC).

POSEIDON skupina 3: Bolnice, mlajše od 35 let z zmanjšanim odzivom jajčnikov (AMH<1.2ng/mL, AFC<5).

POSEIDON skupina 4: Bolnice, starejše od 35 let z zmanjšanim odzivom jajčnikov (AMH<1.2ng/mL, AFC<5).

Pri vseh bolnicah bomo uvedli enoten protokol s spodujanjem jajčnikov v folikularni in lutealni fazi menstruacijskega ciklusa. Enaindvajseti dan ciklusa bomo uvedli 4 mg estradiol valerata za sinhronizacijo in koordinacijo rasti foliklov. Na dan 2 menstruacijskega ciklusa bomo prekinili z estradiol valeratom in pričeli spodbujanje z zdravili (rFSH 300 IU/dan). Z antagonisti gonadoliberinov bomo pričeli 7. dan ciklusa, tako v folikularni kot v lutealni fazi. Za zorenje jajčnih celic bomo uporabili GnRH agonist (Gonapeptyl 0.1 mg sc) in sicer takrat, ko folikli dosežejo velikost 17-18 mm v premeru. Aspiracijo foliklov bomo opravili 36 ur po vnosu agonista GnRH. Pet dni po prvi aspiraciji foliklov bomo pričeli spodbujanje na enak način kot v folikularni fazi. Oploditev, gojenje do blastociste in zamrzovanje zarodkov bomo izvedli po ustaljenih laboratorijskih metodah. Blastociste bomo po odmrznenju zarodkov prenašali v naslednjem spontanem ali spodbujenem ciklusu. Gojišča, v katerih bomo gojili zarodke, bomo shranili na -80 st, za kasnejšo neinvazivno PGT-A. Novo metodo bomo razvili in najprej potrdili na kliničnem PGT programu. Na ta način bomo lahko ločeno ugotovili kakšen vpliv ima sluznica maternice oz. kvaliteta zarodkov na stopnjo zanositev po postopkih zunajtelesne oploditve bolnic z zmanjšanim odzivom jajčnikov.

Neinvazivno metodo določitve kromosomskega statusa zarodka po izolaciji DNK iz gojišča, v katerem bo zarodek gojen, bomo najprej validirali pri zarodkih v rednem kliničnem PGT programu. Šele nato bomo z neinvazivnim pristopom analizirali kromosomski status zarodkov pri naši ciljni populaciji bolnic z zmanjšanim odzivom jajčnikov. Natančneje to pomeni, da bomo zarodke v PGT-SR programu gojili na enak način, kot jih tudi sicer gojimo v klinični praksi (3. dan predimplantacijskega razvoja individualno gojenje v kapljicah z volumnom 40 mikrolitrov). Na ta dan z laserjem naredimo odprtino v ovojnici zarodka - tako se blastocista lahko lažje levi in zato tudi lažje biopsiramo fragment trofoblasta za genetsko analizo. Za primerjavo z invazivnim postopkom bomo tik pred biopsijo odvzeli 10 mikrolitrov gojišča, v katerem se zarodek goji in ga nato genetsko testirali. Po potrjeni zanesljivosti rezultatov, bomo uporabili enako metodo pri naši ciljni populaciji bolnic. Zarodke teh bolnic bomo gojili popolnoma enako kot sicer. Edina razlika bo le v tem, da bomo te zarodke od 3. dneva naprej gojili individualno v kapljicah gojišča. Na 3. dan bomo zarodke tudi temeljito sprali s svežim gojiščem, z namenom da bi z njihove površine odstranili morebitne ostanke DNK granuloza celic ali spermijev, ki bi lahko v analizi dali popačene podatke.

Predvidevamo, da pri bolnicah z zmanjšano rezervo jajčnikov na nizko stopnjo zanositev vpliva tako zmanjšano število pridobljenih zrelih jajčnih celic z večjim številom zarodkov z nepravilnim številom kromosomov, kot tudi spremenjena receptivnost maternične sluznice, zato bomo za oceno receptivnosti določali profil izražanja proteinov v sluznimi maternice v času predvidenega vgnezditvenega okna. Pri ženskah z dvojnim spodbujanjem jajčnikov bomo vzorec sluznice in izpirek maternične votline odvzeli pred vstopom v postopek zunajtelesne oploditve, v obdobju implantacijskega okna in enak postopek ponovili 5 dni po prvi aspiraciji foliklov oz. neposredno pred pričetkom spodbujanja v lutealni fazi. To je čas, primeren za prenos zarodkov v svežih postopkih zunajtelesne oploditve. Vse vzorce bomo zhranili pri -80 st za kasnejšo hkratno analizo. Pri izvajanju dvojnega spodbujanja bomo pridobili informacijo, kako se profil proteinov v sluznici v času vgnezditvenega okna spontanega menstruacijskega ciklusa razlikuje od profila proteinov v sluznici po spodbujanju z visokimi odmerki gonadotropinov, kar lahko posledično vpliva na vgnezditev in razvoj zarodka. Profil proteinov bomo določali s proteinskimi mrežami, ki omogočajo visoko zmogljive tarčne študije večjega nabora proteinov v različnih bioloških vzorcih. Profila proteinov v sluznici zaradi visoke cene ne bomo mogli opraviti pri vseh preiskovankah. Predvidevamo, da bomo lahko določili vgnezditveno okno na osnovi sklepanja iz analize profila proteinov pri 30 bolnicah. Iz POSEIDON skupine 1 bomo opravili odvzem petim bolnicam iz podskupine 1a in petim bolnicam iz podskupine 1b. Na enak način bomo opravili odvzem tudi bolnicam iz skupine 2. Iz skupine 3 in 4 pa bomo opravili odvzem petim ženskam iz vsake skupine. Pri vsaki bolnici bomo z mikromrežami analizirali proteine 2 krat, in sicer v spontanem in spodbujenem ciklusu.

Z neinvazivno metodo določitve kromosomskega statusa zarodka po izolaciji DNK iz gojišča (PGT-A), v katerem bo zarodek gojen, bomo v sodelovanju z genetiki poskusili vzpostaviti novo metodo določevanja kromosomskega statusa blastocist. Metoda je tudi v svetu še v razvijanju in preverjanju. Prednost predvidene metode v primerjavi z biopsijo trofektoderma je ta, da se izognemo možni poškodbi zarodka. Selekcija blastocist bi temeljila na analizi pomnožene DNK v gojišču.

Primarni izidi raziskave bodo:

- Število jajčnih celic
- Število zrelih jajčnih celic
- Stopnja oploditve
- Število zarodkov
- Število zarodkov visoke kakovosti
- Stopnja zanositve

Vse navedene izide (1-6) bomo ocenjevali s primerjavo rezultatov kontinuiranega spodbujanja s prejšnjima postopkoma ZTO skupaj.

- Razlika v profilu proteinov v maternični sluznici med spontanim in spodbujenim ciklusom
- Razlika v profilu proteinov v maternični sluznici med ženskami, ki bodo zanosile in tistimi, ki ne bodo zanosile.

Sekundarni izidi raziskave:

- Delež zarodkov z nepravilnim številkom kromosomov pri pacientkah z zmanjšanim odzivom jajčnikov, mlajših od 35 let
- Delež zarodkov z nepravilnim številkom kromosomov pri pacientkah z zmanjšanim odzivom jajčnikov, starejših od 35 let
- Povprečna poraba gonadotropinov na eno jajčno celico v prejšnjih dveh opravljenih postopkih zunajtelesne oploditve v primerjavi s kontinuiranim postopkom.

7. Ocena etičnih vidikov raziskave

Menimo, da je raziskava etično neoporečna, ker bomo v poteku zagotavljali sledeče:

- vključevanje pacientk v raziskavo bo prostovoljno in tudi pri preiskovankah, ki bodo sodelovanje zavrnile le-to ne bo vplivalo na potek njihovega zdravljenja;
- odvzem vzorcev krvi za določitev koncentracije hormonov (AMH), meritev števila antralnih foliklov (AFC), stimulacija jajčnikov in aspiracija foliklov so del rutinskih in dobro uveljavljenih diagnostičnih in terapevtskih postopkov;
- zamrzovanje zarodkov je del rutinskih postopkov OBMP in ne predstavlja večjega tveganja za vitalnost zarodkov;
- prenos odmrznjenih zarodkov ne predstavlja tveganja za zmanjšanje stopnje zanositve, saj je že dokazano, da je stopnja zanositev s svežimi ali odmrznjenimi zarodki primerljiva;
- odvzem izpirka maternične votline in biopsija endometrija sta neboleča postopka, ki ne predstavljata nikakršnega tveganja za pacientko in ne vplivata na uspešnost postopkov zdravljenja neplodnosti;
- določitev kromosomskega statusa zarodka po izolaciji DNK iz gojišča (PGT-A) je neinvazivna metoda in ne predstavlja tveganja za vitalnost zarodkov.

8. Ali bodo udeležencem dostopni podatki o njihovem zdravju in rezultati raziskave?

Preiskovanke, ki bodo želele biti obveščene o rezultatih raziskave, bodo lahko pisno zaprosile za kratko poročilo, ki bo ob koncu posamezne faze raziskave pripravljeno posebej za njih. V kolikor bodo želele izvedeti rezultate lastnih vzorcev jim bomo posredovali rezultate v primerni obliki.

9. Varnost oseb v raziskavi in varovanje zaupnosti osebnih podatkov

Osebe v raziskavi bodo obravnavane po enakih, splošno sprejetih principih zdravljenja neplodnosti. Preiskovanke lahko kadarkoli, brez obrazložitve, izstopijo iz raziskave. Izstop iz raziskave ne bo vplival na nadaljno zdravstveno obravnavo. V kolikor bo pri preiskovankah prišlo do suma na zaplet (bolj verjetno zaradi utečenih postopkov zdravljenja, kot zaradi modifikacij za namen raziskave) bodo preiskovanke lahko kontaktirale odgovorno osebo prof. dr. Edo Bokal Vrtačnik, dr.med na telefonsko številko: 01/522-60-60.

Modifikacija obstoječega protokola spodbujanja jajčnikov za ciljno populacijo žensk z ZOJ predstavlja minimlane možnosti tveganja za zdravje preiskovank. Najpogostejši zaplet ZTO je sindrom hiperstimulacije jajčnikov, ki pa je v naši tarčni populaciji preiskovank praktično nemogoč, saj gre za dokazano zmanjšano odzivnost jajčnikov na stimulacijo. Lahko so prisotna druga tveganja, podobno kot pri vseh klasičnih postopkih ZTO, ki predstavljajo zaplete zaradi krvavitve ali vnetja ob invazivnem postopku aspiracije foliklov. Vsi sodelujoči v raziskavi so te zaplete sposobni prepoznati in so zmožni pravilno ukrepati pri zdravljenju le-teh. KO za reprodukcijo omogoča tako ambulantno, kot hospitalno (medikamentozno in operativno) zdravljenje zapletov.

Varovanje osebnih podatkov v raziskavo vključenih oseb bomo izvajali s pomočjo posebnega kodiranega označevanja vzorcev, iz katerega ne bodo razvidni osebni podatki preiskovank. Baza podatkov bo shranjena pri odgovorni raziskovalki prof. dr. Edi Bokal Vrtačnik, dr.med. in ne bo dostopna drugim.

Raziskovalci se zavezujemo, da se bomo držali načel Helsinške deklaracije o biomedicinskih raziskavah na človeku, Konvencije Sveta Evrope o varovanju človekovih pravic in dostojanstva človeškega bitja v zvezi z uporabo biologije in medicine (Oviedske konvencije) in načel slovenskega Kodeksa zdravniške etike.

Raziskava bo sicer potekala na ženskah v rodni dobi, vendar brez povečanega tveganja teratogenosti, zmanjšane plodnosti ali nevarnosti ob morebitni nosečnosti. V primeru, da bi v raziskavi ugotovili pomembne podatke, ki bi bili terapevtsko in/ali prognostično pomembni za preiskovanke, bomo udeležene v raziskavi o tem obvestili in primerno poučili.

10. Plačnik raziskave

Zdravniki raziskovalci za svoje sodelovanje v raziskavi ne bodo deležni finančnega nadomestila. Zdravljenje neplodnosti je del redne klinične obravnave in za to ne bodo potrebna dodatna finančna sredstva. Dodatna sredstva za analizo izpirkov maternične votline in bioptatov endometrija pa bomo zagotovili iz sredstev za terciarni projekt UKC Ljubljana. Vsi dodatni raziskovalni postopki bodo opravljeni v breme raziskovalnega časa in denarja.

11. Priloge

- Izjava odgovorne raziskovalke Ginekološke klinike UKC Ljubljana
- Izjava predstojnice Kliničnega oddelka za reprodukcijo UKC Ljubljana
- Izjava strokovnega direktorja Ginekološke klinike UKC Ljubljana
- Izjave sodelujočih v raziskavi
- Izjava predstojnika Kliničnega inštituta za genomsko medicino
- Pisne informacije o raziskavi za preiskovanke in obrazec izjave o zavestni in svobodni privolitvi sodelujočih
- Potrdilo o plačilu takse
